# Steroidal saponin profiles and their key genes for synthesis and regulation *in Asparagus officinalis* L. by joint analysis of metabolomics and transcriptomics

**DOI:** 10.1186/s12870-023-04222-x

**Published:** 2023-04-20

**Authors:** Qin Cheng, Liangqin Zeng, Hao Wen, Sylvia E. Brown, He Wu, Xingyu Li, Chun Lin, Zhengjie Liu, Zichao Mao

**Affiliations:** 1 College of Agronomy and Biotechnology, Yunnan Agricultural University (YNAU), Kunming, 650201 Yunnan China; 2 Institute of Improvement and Utilization of Characteristic Resource Plants, YNAU, Kunming, China; 3 The Laboratory for Crop Production and Intelligent Agriculture of Yunnan Province, Kunming, China

**Keywords:** *Asparagus officinalis*, Steroidal saponins biosynthesis, Transcriptomics, Metabolomics

## Abstract

**Background:**

*Asparagus officinalis* L. is a worldwide cultivated vegetable enrichened in both nutrient and steroidal saponins with multiple pharmacological activities. The upstream biosynthetic pathway of steroidal saponins (USSP) for cholesterol (CHOL) synthesis has been studied, while the downstream pathway of steroidal saponins (DSSP) starting from cholesterol and its regulation in asparagus remains unknown.

**Results:**

Metabolomics, Illumina RNAseq, and PacBio IsoSeq strategies were applied to different organs of both cultivated green and purple asparagus to detect the steroidal metabolite profiles & contents and to screen their key genes for biosynthesis and regulation. The results showed that there is a total of 427 compounds, among which 18 steroids were detected with fluctuated concentrations in roots, spears and flowering twigs of two garden asparagus cultivars. The key genes of DSSP include; steroid-16-hydroxylase (S16H), steroid-22-hydroxylase (S22H) and steroid-22-oxidase-16-hydroxylase (S22O-16H), steroid-26-hydroxylase (S26H), steroid-3-β-glycosyltransferase (S3βGT) and furostanol glycoside 26-O-beta-glucosidases (F26GHs) which were correlated with the contents of major steroidal saponins were screened, and the transcriptional factors (TFs) co-expressing with the resulted from synthetic key genes, including zinc fingers (ZFs), MYBs and WRKYs family genes were also screened.

**Conclusions:**

Based on the detected steroidal chemical structures, profiles and contents which correlated to the expressions of screened synthetic and TFs genes, the full steroidal saponin synthetic pathway (SSP) of asparagus, including its key regulation networks was proposed for the first time.

**Supplementary Information:**

The online version contains supplementary material available at 10.1186/s12870-023-04222-x.

## Background

Asparagus (*A. officinalis* L.), also called garden asparagus, which has been cultivated worldwide for thousands of years, is known as the king of vegetables not only for its enrichened nutrient components, such as essential amino acids, vitamins, and minerals but also for the accumulation of bioactive medicinal compounds, such as steroids and flavonoids [1]. Asparagus steroids and their derived saponins are regarded as major active pharmacological compounds for antitumor, antifungal and modulating cytotoxic activities [2]. It was reported that steroidal saponins from different cultivars of asparagus could inhibit the growth of multiple human cancer cell lines [3]. The ethanol extracts from asparagus young stem (spear) and root have accumulated steroidal compounds and are found to have activities of anti-inflammatory, antibacterial and antivirus. They could also be used to control diabetes, hyperlipidemia and heat shock protein 70 (HSP70) mediated redox imbalance. Some isolated single purified steroidal compound has been found to obviously have antitumor activities in both in vitro and in vivo [4, 5].

In the steroidal saponins biosynthesis pathway (SSP), CHOL is regarded as the key inter metabolite. Although the biosynthetic pathway of CHOL has been elucidated in model plants [6], there are only a few reports on further modification of CHOL, which is proposed as the downstream of SSP(DSSP). Mohammadi et al. proposed that the biosynthetic pathway of diosgenin in *Trigonella foenum‐graecum* L*.* consists of several catalytic steps including successive hydroxylation and/or oxidation of cholesterol on C22, C16 and C26 positions, followed by the closing of the E- and F-rings of steroidal skeletons with different type hydroxylases and/or oxidases [7]. With similar earlier catalytic steps of CHOL modification, the biosynthesis of steroidal glycoside alkaloids was partially elucidated in *Veratrum californicum*, potato (*Solanum* *tuberosum*) and tomato (*Solanum lycopersicum*) [8, 9]. The key catalytic steps of DSSP in both *Trigonella foenum‐graecum* L. *and Paris polyphylla*, which were elucidated by Christ et al., showed that PpCYP90G4/TfCYP90B50 catalyzed hydroxylation of both C22 and C16 positions of CHOL respectively, and further oxidation of C22-OH to ketone of steroidal skeletons, followed by E ring formation, and C26 hydroxylation catalyzed by PpCYP94D108/PpCYP94D109/ PpCYP72A616 of *P. polyphylla* or TfCYP82J17/TfCYP72A613 of *T. foenum‐graecum* L. to form diosgenin with closing F-rings of steroidal skeletons [10]. However, a recent study by Zhou et al., suggested a different pathway in the steroids biosynthetic pathway of *Dioscorea zingiberensis,* in which CHOL was first catalyzed to 22R-OH-CHOL by DzCYP90B71(S22H), followed by DzCYP90G6 ( S22O-16H) catalyzing both the hydroxylation of C16 and the oxidation at C22R-OH to C22-Keto of steroids, then both C22-keto and C16-OH linked to form the E-ring of steroidal skeletons; after which, the C26 position is hydroxylated by DzCYP94N8 or DzCYP94D43 and closing with C22-keto and C26-OH to form the F-ring of steroidal skeletons [11]. These studies provided good cases on steroidal saponins synthesis in the plant. However, the synthesis and regulatory genes for steroidal compounds acumination for plant survival and environmental adaptation in various species of flowering plants, including asparagus, remains unknown.

For studying steroids biosynthesis in asparagus, Yi et al. identified the genes in the early steroidal saponin synthetic steps of asparagus, which involve the biosynthesis of protodioscin which is the main saponins components in the edible parts of spears by using the de novo assembled transcriptome of both green and white spears of asparagus [12]. As a result of not using the available genome of asparagus, the key genes and transcription factors related to biosynthesis, especially the genes of DSSP for the biosynthesis of major garden asparagus steroidal compounds such as diosgenin, dioscin, trillium and other spirostatic saponins have not been documented, therefore, it is still necessary to analyze the contents and profiles of steroidal saponins and to screen their key synthetic and regulatory genes with the published genome of *A. officinalis* L*.* [13].

In this study, both the transcriptomic and metabolomic strategies were applied to green and purple asparagus organs with cultivars of “Guelph Millennium” and “Purple Passion” respectively, to analyze the profiles, distribution and steroidal contents, to screen key genes and regulatory transcription factors of steroidal saponins biosynthesis to propose the full biosynthetic pathway with its regulation networks in garden asparagus.

## Results and analysis

### The total steroids contents, metabolite profiles and obtained RNAseq data

**Fig. 1:**
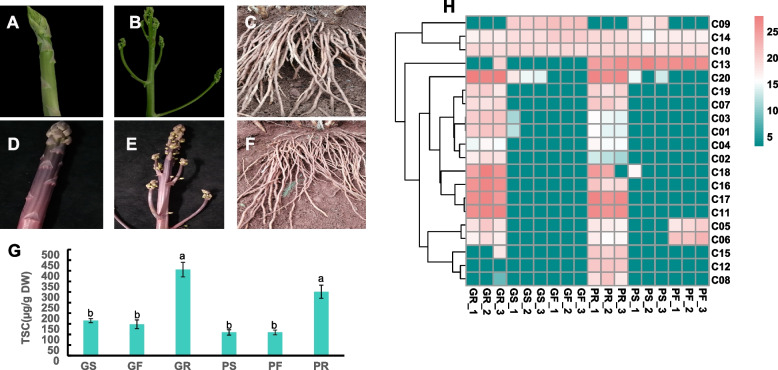
Steroidal metabolites in both asparagus cultivars; **A** ~ **F**, samples of spears (Ss), flowering twigs (Fs) and roots (Rs) of green (G) and purple (P) asparagus, respectively; **G** total steroidal saponins content (TSC) in Ss, Fs and Rs of green and purple asparagus; **H** abundance heatmap of steroid and terpenoid metabolites detected by liquid chromatography-mass spectrometry (LC–MS); note, the names and structures of corresponding assigned chemical numbers (C01 ~ C20) were showed in Fig. S1

The total steroidal saponins contents (TSCs) in the roots (Rs), spears (Ss) and flowering twigs (Fs) of both green and purple asparagus were determined and the results showed that there was no significant difference between the same organs of cultivars. However, the TSCs of Rs in both cultivars of asparagus were significantly higher than that of the Ss or Fs respectively (Fig. 1G). A total of 437 metabolites were detected in 18 samples, including 2 terpenoids (C09, C13) and 18 steroids (including cholesterol(C10), 3β-alcohol-5-β progesterone-16-ene-20-one-3-O-a-L-arabinopyranosyl(C14), 4 furostane saponins (C06, C07, C18, C19), 7 isospirostane saponins (C01 ~ 05, C08, C11), and 6 spirostane saponins (C12, C15 ~ 17, C20) (Fig. S1). Furthermore, most steroidal compounds were accumulated with a higher content in Rs which is demonstrated by the heatmap of metabolites abundance (Fig. 1H). The results also showed that C05 and C06 are highly accumulated in Rs of green and Fs of purple asparagus cultivars, while C08, C12 and C15 only have higher concentrations in Rs of purple asparagus cultivars (Fig. 1G, Fig. S1).

The statistics of RNA-seq data were taken and shown in Table S1 to evaluate the quality of sequencing, which includes data size and quality, reads length and mapping ratio, and the results showed the quality of RNAseq was nice. The raw reads of insertion (ROI) were processed with IsoSeq3 pipelines to get 1.6, 0.6 & 1.0 Gb circular consensus sequence (CCS) with an average length of 2.4, 2.0 & 1.7 kb and a mean number of passes of 32, 24 & 25 respectively (Table S2). The resulting Pacbio RNAseq data were used for optimizing annotation of the detected synthetic and regulatory genes of SSP in asparagus, and detection of their possible exon alternative splicings (ASs) and genes fusions.

### Analysis of differential metabolites accumulation and correlated genes expression among organs and between asparagus cultivars

**Fig. 2:**
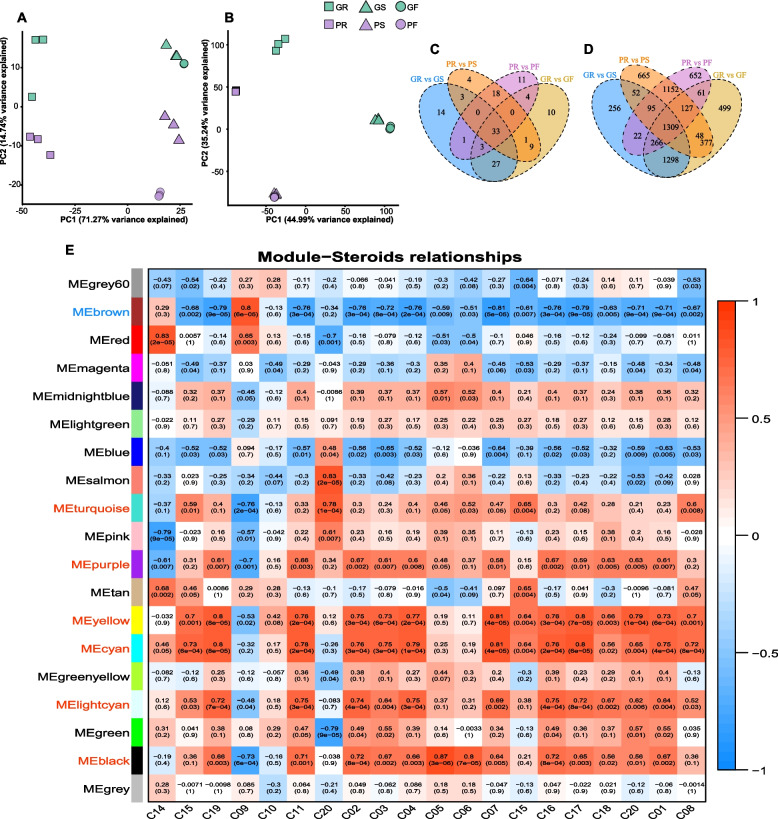
The steroids profiles and the genes related to steroids biosynthesis, **A**, the principal component analysis (PCA) of steroids and terpenoid metabolites in green and purple asparagus cultivars based on the abundance; **B**, the PCA of all genes in green and purple asparagus based on FPKM matrix; **C** and **D**, the venn diagrams of detected up-regulated differential metabolites (UDMs) and up-regulated differentially expressed genes (UDEGs) in GRs vs GSs, GRs vs GFs, PRs vs PSs, PRs vs PFs, respectively; **E** the correlation among WGCNA gene modules and the steroid metabolites with highlighting target modules with red (positive correlation) or blue (negative correlation)

The principal component analyses (PCAs) were used for both metabolite abundances and all gene expression analyses. PCA results of gene expression (FPKM) from Illumina RNAseq data showed a similar clustering pattern among Rs, Ss and Fs of both green and purple asparagus cultivars, in which Ss and Fs clustered together within both green and purple asparagus samples, respectively. However, they have separated distribution between the two cultivars. Moreover, GRs and PRs themselves were clustered in one direction with obvious separation, and Rs are significantly different from all Ss and Fs samples, respectively (Fig. S2A, Fig. 2A, B). In addition, the PCA results of all detected steroidal metabolites (Fig. 2A) also showed a similar clustering pattern as well, indicating the detected metabolites (including steroids) biosynthesis and accumulation were closely correlated to the detected expression genes of metabolism and regulation of those metabolic compounds. The TSCs (Fig. 1G) and steroid metabolites profiles (Fig. 1H) in detected samples showed that there are greater differences in steroids content and profiles between Rs vs. Ss and Rs vs. Fs, and Ss vs. Fs within the green and purple asparagus cultivars, while Rs, Ss and Fs between green and purple cultivars show significant differences (Fig. 2). These PCA results suggest there were similar expression patterns of SSP genes within and between organs and cultivars of both green and purple asparagus. Therefore, the differential metabolites (DMs) and differential expression genes (DEGs) analyses of the four groups, GRs vs. GSs, GRs vs. GFs, PRs vs. PSs, and PRs vs. PFs, were selected to perform further analyses. The Venn diagrams of DMs and DEGs from the above 4 groups showed that 104 metabolites and 3,303 genes were intersected for both metabolites contents and genes expression, which were differential between Rs vs. Ss and Rs vs. Fs (Fig. S2 F, G), in which 33 up-regulated differential metabolites (UDMs) and 1309 up-regulated differentially expressed genes (UDEGs) with higher contents and higher expression in Rs were selected for further analyses (Fig. 2 C, D).

For analyzing the diffident metabolites and gene expression of the same organs between green and purple asparagus cultivars, GRs vs. PRs, GSs vs. PSs, and GFs vs. PFs of both Illumina RNAseq reads and metabolic compounds were also conducted for their DEGs and DMs analyses. The Venn diagram of DMs and DEGs from the above 3 groups showed that 23 metabolites and 3174 genes were intersected for both metabolite contents and genes expression with differences among GRs vs. PRs, GSs vs. PSs, and GFs vs. PFs (Fig. S2 E). The results showed that both the DEGs of GRs vs. PRs and GSs vs. PSs were only enriched in the pathway phenylpropanoid biosynthesis. While the DEGs of GFs vs. PFs were enriched in not only pathways of phenylpropanoid biosynthesis but also the biosynthesis of the steroid-related pathway, steroids hormone biosynthesis &signaling transduction and second metabolite modification catalyzed by CYP450 family genes as well(Fig. S2 B ~ D) The results were consistent with the different phenotypes of green and purple colors of their spears and different nutritional components detected by metabolomics analysis in Fs and Ss of the cultivars, respectively (Fig. 1G and Fig. S2F, G).

**Fig. 3:**
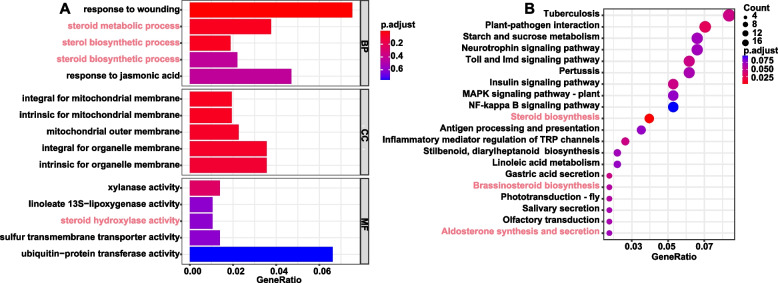
The GO and KEGG [41] enrichment analyses of intersected genes of both UDGEs and WGCNA gene modules correlated with steroidal metabolites; **A**, GO enrichment analysis with highlighting steroid-related terms in red; **B**, KEGG enrichment analysis with highlighting steroid-related paths in red as well

To fully screen the key genes of the biosynthesis and regulation of steroidal metabolites, except the above DGE analysis method, the alternative strategy with co-expression genes modules correlated steroidal compounds contents was conducted by WGCNA as well. A total of 18 samples with gene expression matrix normalized by FPKM were used to conduct co-expression analyses. The results showed that all the expressed genes were clustered into 19 co-expression genes modules labeled with different colors (Fig. S3), and the genes module which correlated with the abundances of 18 steroids and 2 terpenoids was analyzed (Fig. S3 and Fig. 2E). According to the result, 7 modules, including 6 positive and 1 negative, were selected with a critical standard of *p*-value ≤ 0.05 and absolute correlation value ≥ 0.7. Then the genes in the modules positively correlated to steroids were named co-expression genes modules of SSP (SSPGM). To improve the reliability of key genes of SSPGM, the intersection of the UDEGs and SSPMG were merged to obtain 814 genes. Furthermore, GO and KEGG enrichment analyses were conducted using the resulting 814 intersected genes. The GO enrichment results of 814 intersected genes sets showed that the genes were enriched in terms of steroid biosynthesis and responding to wounding etc., in the biological process of GO, while molecular function enriched in steroid hydroxylase activities, which was essential for steroidal saponin synthesis for CHOL modification with *P* value ≤ 0.05 (Fig. 3A). The KEGG enrichment results (Fig. 3B) also showed that steroids and brassinosteroid biosynthesis pathways were enriched. Based on the above enrichment analysis, 29 key candidates of SSP genes were screened, including 12 cytochrome P450s (P450) family genes, 2 glycosyltransferases (GTs) family genes, 3 glucosidases (GHs) genes for DSSP and several CHOL synthesis genes for USSP (Fig. S4 and Fig. S5).

### Identification of the genes involved in USSP of asparagus

Due to strict selections in the above analyses, the above-selected USSP synthetic genes, which may only code for the key steps of catalytic enzymes, were not the complete gene sets of asparagus USSP. Therefore, the protein sequences of enzymes encoded for the cholesterol synthesis genes whose functions have been characterized were downloaded from NCBI (https://www.ncbi.nlm.nih.gov/) or/and UniProt (https://www.uniprot.org/) for homologous searching with BLAST, following the construction of clustering trees (Fig. S4) by MEGAX to obtained possible CHOL synthetic genes sets in asparagus. The results showed that each step of enzymatic genes of CHOL synthesis was predicted with critical standards with protein similarity of 50% and average coverage of 85%. The CHOL synthesis homologous genes of asparagus were obtained, which include 1 acetyl-CoA C-acetyltransferase (AACT), 2 hydroxymethylglutaryl-CoA synthases (HMGSs), 2 hydroxymethylglutaryl-CoA reductases (HMGRs), 2 mevalonate kinases (MVKs), 1 phosphomevalonate kinase (PMVK), 1 diphosphomevalonate decarboxylase (MVD), 2 isopentenyl-diphosphate Delta-isomerases (IDIs), 1 geranylgeranyl diphosphate synthase (GGPPS), 1 farnesyl diphosphate synthase (FPPS), 1 (S)-squalene synthase (SS), 2 (S)-squalene-2,3-epoxide hydro-lyases (SEs), 2 cycloartenol synthases (CASs), 3 sterol side chain reductases ( SSRs), 4 C-4 sterol methyl oxidases (SMOs), 1 cyclopropylsterol isomerase (CPI), 1 sterol C-14 demethylase (CYP51), 2 sterol C-14 reductases (C14Rs), 3 C-4-OH-sterol methyl oxidases (3βHSDs), 2 sterol C-5 (6) desaturase (C5-SDs), and 1 7-dehydrocholesterol reductase (7-DR) (Fig. S4). These asparagus CHOL biosynthetic genes were manually annotated optimized and detected their possible exon alternative splicings (As) and gene fusion (fusion) with RNAseq data obtained from both Illumina and PacBio platforms (Table S3). The results showed that all the USSP genes except a 3βHSD (evm. model. AsparagusV1-06.1107, (06.1107), same as below) have full CDS with transcripts harboring both 5’ and 3’ translational regions (5’UTR and 3’UTR). Additionally, a PMVK (3.1112), a CYP51 (Unassigned.240, (Un.240) same as below), an SMO (07.13), a C14R (Un. 725) have ASs; and a HMGS (5.1543) have fusion, while a βHSD (01.3101) have both fusion and ASs indicting the genes of USSP in asparagus annotated at isoform level and the ASs and Fusions of these genes may have contributed to the regulation of USSP for different synthesis and accumulation of CHOL among organs and between asparagus cultivars (Table S3 and Fig. S4).

### Identification of the genes involved in DSSP of asparagus

**Fig. 4:**
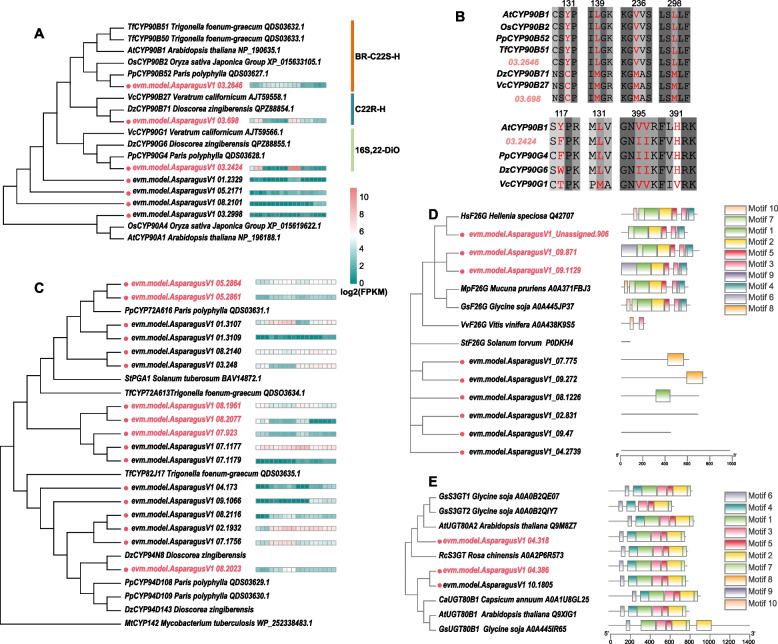
Identification of key genes of downstream of steroidal saponins biosynthetic pathway (DSSP); **A**, the phylogenetic tree was constructed using CYP450s of asparagus and the known CYP90s family genes involved in steroids C22 hydroxylation based on protein sequence similarity by Neighbor-Joining Algorithm (NJ), and the gene name listed as gene symbol, organism name and NCBI access No., respectively, while the asparagus genes showed expression level with the heatmap in GR, GS, GF, PR, PS, and PF with 3 replicates, respectively; **B**, the multiple sequence alignment of three CYP90s genes in asparagus with functionally characterized CYP90s genes, highlighting the conserved amino acid residues in red; **C**, the phylogenetic tree of CYP450s from asparagus and known CYP72s and CYP94s family involved in steroids C26 hydroxylation based on protein sequence similarity by NJ, the gene name listed as above; **D**, the phylogenetic tree was constructed using genes annotated as glucosidase in asparagus and functionally characterized furostanol glycoside 26-O-beta-glucosidase (F26G) genes by NJ, with gene name followed with their protein conserved motifs predicted using MEME. **E**, the phylogenetic tree was constructed using glycosyltransferases (GTs) genes in asparagus and functionally characterized GTs genes by NJ and the motifs were shown as in D

**Fig. 5:**
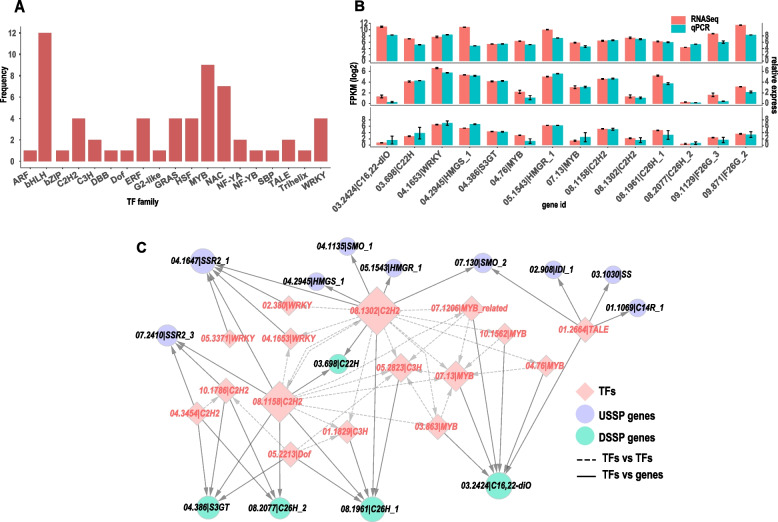
The regulatory network of TFs and genes in SSP in asparagus. **A**, the predicted TFs related to SSP in asparagus; **B**, qPCR analysis of the selected key genes and TFs of SSP in asparagus; **C**, the regulatory network based on gene expressions of TFs and genes of SSP in asparagus, the TFs were represented by prismatic, the genes of cholesterol synthesis named as upstream SSP (USSP) were represented by circular filled with purple, and the genes for cholesterol modification named as downstream of SSP (DSSP) were represented by circular filled with green, and the size showed the connectivity in the network

It is generally believed that CHOL is used as an important intermediate metabolite in the biosynthesis of steroidal saponins, further CHOL modification catalyzed by a series of hydroxylases, oxidases, glycosyltransferases (GTs) and/or glucosidases (GHs) to get the final steroids and steroidal saponins. Therefore, to screen the key hydroxylases genes involved in DSSP, the homologs searching with functionally characterized P450 genes involved in steroidal saponins biosynthesis were performed, and the phylogenetic tree was conducted as well (Fig. 4A ~ C and Fig. S5). The results showed that 3 genes (03.2424, 03.698, 03.2646) of the P450 superfamily from asparagus (*AoCYP450s*) clustered together with the known function of CYP90s from other organisms. Further, multiple protein sequence fine alignment (MSA) showed that 03.2646 was highly conserved in 4 key amino acid residues, which are essential for catalytic activities in AtCYP90B1, a representative of the enzyme catalyzing the CHOL to 22(S)-OH-CHOL for brassinosteroids(BR) biosynthesis in *Arabidopsis thaliana* [14] (Fig. S6); and the 03.698 is highly conservative in those amino acid residues in functionally characterized DzCYP90B71 [11] and VcCYP90B27 [9]. While the 03.2424 was conserved in 5 key amino acid residues with the characterized DzCYP90G6 and PpCYP90G4 playing roles as a bifunctional enzyme of C16 hydroxylase and C22-keto sterol oxidase (S22O-S16H) (Fig. S6 and Fig. S7). Therefore, 03.2646 was referred to as BR biosynthesis-related C22 sterol hydroxylase (BR-S22H), 03.698 to steroidal saponin-related C22R hydroxylase (S22H), and 03.2424 to a bifunctional enzyme of S22O-16H for CHOL modification based on their expression patterns in both green and purple asparagus (Fig. 4A ~ B, Figs. S5, S6, and S7). Based on the structure of detected steroidal saponins, which includes furosteroidal, isospirosteroidal and spirosteroidal saponins, such as pseudoprotodioscin (C06), asparasaponin II (C18), dioscin (C04) and asparanin B (C17) (Fig. S1, Figs. S5, S6, and S7), the glycosyltransferases ( GTs), glycoside hydrolases(GHs) and steroidal C26 hydroxylase (S26H) should play important roles in DSSP as well. Therefore all detected GTs and GHs families genes in both UDEGs and SSPGM intersection set were blasted with known functional enzymes in CAZY database (http://www.cazy.org/Home) to screen candidate furostanol glycoside 26-O-β-glucosidases(F26Gs) and GTs in DPPS of asparagus (Fig. 4D). The results showed that 3 F26Gs (Un.946, 09.871 and 09.1129) and 2 C3 steroids-β-glycosyltransferases (S-3β-GTs, 04.318, 04.386) were selected based on their list in UDEGs and SSPGM sets, but also closely clustered with functionally characterized F26Gs and S3GTs with their conserved amino acid motifs (Fig. 4D, E). By using similar methods, 6 *S26H* (*05.2861*, *05.2864*, *07.923*, *08.1961*, *08.2023*, *08.2077*) were selected (Fig. 4C and Fig. S5). The resulting 2 genes located on chromosome (Chr) 5 (*05.2861*, *05.2864*) clustered to a clade of CYP72A with functional confirmation in *P. polyphylla* (*PpCYP72A616* with NCBI accesses No: QDS03631.1 same as below); and 1 gene located on Chr 8 (*08.2023*) clustered to CYP94D108 with functional confirmation in *P. polyphylla* (*PpCYP94D108*, *QDS03629.1*); while, 3 additional genes (*07.923*, *08.1961* and *08.2077*) located on Chr 7 and Chr 8 respectively have high expression levels in Rs in both cultivars, thus were selected as additional possible candidate genes of *S26Hs* in asparagus as well (Fig. 4C, Fig. 5 and Figs. S5, S6, and S7).

In addition, it was reported that S16DOX and St16DOX belonging to the 2-OGD family members were involved in the steroidal C16 hydroxylation [15] in steroidal alkaloid biosynthesis, so these proteins were used for BLAST and multiple sequence alignments to search for additional steroid C16 hydroxylases in asparagus belonging to the 2-OGD family. 1 gene (*Un.946*), was identified as an additional possible C16 hydroxylase in asparagus (Fig. S8).

### Correlation network of steroidal compounds synthesis regulatory transcription factor (TF) genes

To screen transcriptional factors (TFs) or regulators of steroidal saponins synthesis, all the above-selected intersections of both UDEGs and SSPGM gene were submitted to PlanTFDB (http://planttfdb.gao-lab.org/) for TFs prediction and resulted in 61 TF genes in 19 TF families including 12 bHLHs, 9 MYBs, 4 C2H2 & 2 C3H1 type zinc fingers (ZFs), 4 ERFs, 2 TALEs, 4 WRKYs and 1 DOF (Fig. 5A). Based on transcription factor binding sites (TFBSs) prediction in the promoters, which are 2000 bp upstream flanking from the start coding (ATG) of the SSP synthetic or regulatory genes, respectively (Fig. S9). The TFs and steroids synthetic genes promoter with corresponding TFBSs were regarded as regulation pairs, correlation analysis of expression (FPKMs) between TFs and synthetic genes pairs was conducted based on Pearson correlations with R package of psych [16], and the resulting correlation network was visualized by Cytoscape [17] (Fig. 5C). The results showed that 16 TFs, 9 genes involved in 7 catalytic steps of USSP and 5 genes involved in 4 catalytic steps of DSSP were found to have a positive correlation in the network (≥ 0.8 & *p*-value ≤ 0.05) (Fig. 5A). In detail, the USSP genes (*07.2410*, *04.1647*, *04.2945, 04.1135*, *05.1543*, *07.130*), are correlated with C2H2 zinc finger families, and the gene of *04.1647* was correlated with 3 WRKY families, while genes of *07.130*, *02.908*, *03.1030* and *01.1069* were correlated TALE families [18]. While the DSSP genes seem to be related to multiple TFs; *03.698* (S22H) is regulated by 2 C2H2(08.1158 and 08.1302); *04.386* (S-3βGT) is correlated with 3 C2H2 type ZFs (04.3454, 10.1786, 08.1158) and 1 Dof (05.2213); *08.196* (S26H) is correlated by 1 Dof (05.2213), 2 C3H type ZFs (01.1829, 05.2823) and 1 C2H2 type ZF (08.1302); while *03.2424* (S22O-16H) is correlated by 5 MYBs (03.863, 07.13, 07.1206, 0.1562, 04.76) and 1TALE (01.2664). These correlated TFs in the network may be important for the regulation of synthetic genes in SSP, especially the DSSP. Further detailed cis-element prediction results (Fig. S8 and Table S4) showed that ~ 21 kinds of cis-elements were predicted, including 141 ABREs (involved in the abscisic acid responsiveness), 112 MYCs (motifs responding to chilling), 96 STREs (stress response element), 70 CGTCA-motifs (involved in the MeJA responsiveness), 55 as-1(salicylic acid- and auxin-responsive element), 55 TGACG-motifs (involved in the MeJA-responsiveness), 53 AREs (antioxidant response element related to the anaerobic environment), besides these, EREs (ethylene-responsive element), WRE3 and WUN-motifs (responsible wound element), DREs (dehydration-responsive element), GARE-motifs (gibberellin-responsive element) were also found in both TFs and synthetic genes of SSP in asparagus (Fig. S9 and Table S4). These results suggest that the cis-elements in both synthetic and TFs genes of SSP might be regulated by stresses including cool, wound and drought, and the stress signaling integrated into phytochrome including abscisate (ABA), ethylene (ETH), salicylate (SA) and jasmonate (JA) and GA signaling network to induce steroidal compounds production to respond to environmental stress stimuli for adaptation and survival of asparagus.

To confirm the key synthetic and regulatory genes expression of SSP in asparagus from RNASeq data, 14 genes including TFs coding genes were selected for qRT-PCR amplification to conduced gene expression analysis in different organs from purple asparagus with primers listed in Table S3. The results showed that the expression patterns of both RNAseq and qRT-PCR analysis were nearly consistent (Fig. 5B) indicating the correctness of gene expressions detected from RNAseq data. The PacBio RNASeq data were further used to optimize the annotation of the predicted DSSP genes and all correlated TFs with both 5’UTRs and 3’UTRs. Further ASs and fusions detection of DSSP-related genes found *09.871* encoding F26GH, *03.698* coding an S22H, and a correlated *04.3454* encoding TF of *C*2H2 type ZFs were detected with gene fusion; while the *09.871*gene have alternative splicing as well. It is interesting to find that the DSSP-related genes have less exon alternative splicing and gene fusion than the genes found in USSP (Table S5 and Fig. S10), indicating gene fusion and AS of SSP play important roles in steroidal compounds synthesis and accumulation, especially the genes of USSP for CHOL biosynthesis.

### Structural optimization of screened genes and TFs by isoseq data

The screened genes, 16 TFs and 45 structural (synthetic)genes, were further annotated optimizely by using Pacbio RNASeq data with both 5’ and 3’ UTRs, followed by the possible gene fusion and splicing detection. 8 genes of SSP in asparagus were detected with fusion, and 26 genes were detected with the alternative poly A splicing including important DSSP genes; 03.698, 04.386, and 09.871(Fig. S10 and Table S5). The results suggest that the SSP genes of asparagus were annotated at the transcriptional isoform level with more reliable confidence. The fusion and alternative splicing genes may participate in SSP regulation and result in diversified steroidal saponins accumulation.

### The proposed full steroidal saponins biosynthetic pathway in asparagus

**Fig. 6:**
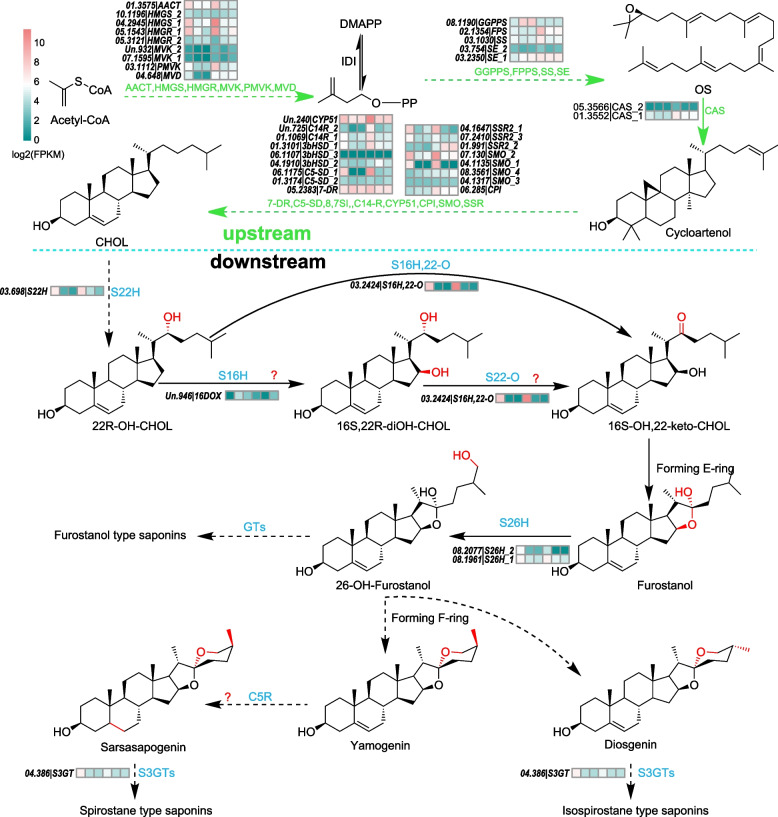
The hypothetical main biosynthetic pathway of steroidal saponins in asparagus. the USSP genes were depicted in green and DSSP genes were marked in blue, besides, the average expression (R, S and F of green and purple asparagus, respectively) were shown in the heatmaps. The one-step reactions were represented with solid lines while the dash lines represented multi-step ones, and the arrows with the question marker represented the less possible step(s). The changes in each reaction on metabolites were highlighted in red in the structural formula

The biosynthesis pathway of steroidal saponins in asparagus was inferred including the key steroid metabolites, their synthesis genes and regulatory TFs as well (Fig. 6). The biosynthesis of steroidal saponins of asparagus can be simply divided into USSP (de novo synthesis of cholesterol) and DSSP (steroid skeleton modification). It is believed that the USSP is the synthesis of CHOL through the MVP pathway in the cytosol, and the genes encoding corresponding enzymes of each step of CHOL are clear in model plants. In this study, steroids including CHOL, a progesterone derivative, 4 furostanol saponins and 13 spirostane or isospirostane saponins were detected (Fig. 1H and Fig. S1), suggesting that the DSSP of asparagus is the multi-branched pathway to synthesis these detected steroids starting from the CHOL modification. The de novo synthesis of CHOL in USSP is catalyzed by many enzymes, including AACTs, HMGSs, HMGRs, MVKs, PMVKs, MVDs, IDIs, GPPSs, FPPSs, SSs, SEs, CASs, SSRs, SMOs, CPIs, CYP51s, C14Rs, 8,7-SIs, C5-SDs, 7-DRs, etc. as described in model plants [6], while the DSSP starts with 03.698 catalysts CHOL to 22R-OH-CHOL; then form 22-keto-16-OH CHOL catalyzed by 03.2424; there is also another possible catalytic pathway: a steroidal C16 hydroxylase of 2-OGD family catalysts 22R-OH-CHOL to 16S,22R-diOH-CHOL, then catalyzed to 22-keto-16-OH-CHOL by S22O-16SH e.g., 03.2424 or unknown oxidase(s). The 22-keto-16-OH-CHOL is unstable and may be able to cyclize to form furostanol spontaneously or catalyzed by the unknown enzyme(s). Then the C26 of the steroidal skeleton is hydroxylated by 08.2077 or 08.1961(S26Hs) to produce 26-OH-Furostanol, and the C26- hydroxyl (OH) group of 26-OH-Furostanol can be further modified by GTs/ GHs to form a glycosidic bond to produce furostanol-type saponins. The modified glycosyl group can also be broken by glycoside hydrolases to form C26-OH and again to form other saponins. C26-OH and C22-OH of Furostanol can be oxidized at first, then cyclize to produce two chemical isomers of yamogenin and diosgenin, respectively. The former is further modified by C5R to produce sarsasapogenin, which is the steroidal aglycone of 5 saponins which were detected in the metabolites data sets. After that, the 3β-OH group of all saponins is modified with one more sugar group by GTs including S3GTs to get different types of steroidal saponins in asparagus. The sugar group modification will increase the solubility of steroidal saponins in water leading to high concentrations in asparagus organs, especially in Rs to respond to abiotic and biotic stresses for adaptation to the environment for perennial survival.

## Discussion

The steroidal compounds profiles and contents in metabolic were consistent with the TSCs determined with the spectrophotometric method. However, C10(Cholesterol) and C14(3β-alcohol-5-β progesterone-16-ene-20-one-3-O-a-L-arabinopyranosyl) showed a high abundance in all three detected organs, which may be due to CHOL being an important component of cell membranes and being the precursor for synthesizing brassinosteroids (BRs) as well [19], while the latter (C14), which has endocrinologic modulation in pregnancy with fascinating immunomodulatory capabilities [20], has a high accumulation in all detected organs of asparagus cultivars, however, have unknown functions and mechanisms.

Previous studies ADDIN EN.CITE [12, 21] found higher contents of protodioscin in white spear than the green spear, and some genes related to which were up-regulated expressed in white spear [12]. In our study, the saponins including protodioscin were mainly detected in roots of green and purple asparagus with varying abundance, but few in stems and flowering twigs, and the most DEGs between the same tissues of green and purple asparagus were enriched in pathways related to flavonoids (Fig. S2 B ~ C). The results mean the difference in saponins and flavonoids between both cultivates. Besides, asparanin A and yamogenin reported as part of the main saponins in green and white asparagus [21] were not detected in this study, while diosgenin and sarsasapogenin, as well as many saponins derived from them, were found with higher abundance in green than purple asparagus. The possible reasons are that, on the one hand, different varieties of asparagus contend different types and contents of saponin; on the other hand, the production mode, such as harvesting time, light or dark, may affect the growth and development to further influence the generation and distribution of metabolites including steroidal saponins to face the changes of environment.

In this study, the key genes of SSP in asparagus, especially, P450 genes of DSSP were identified by combining transcriptome, metabolome and other analyses. In addition, a CYP90B gene related to BR synthesis, 03.2646, was also screened. Interestingly, *Trigonella foenum–graecum* (dicotyledon), *Paris polyphylla* and *Dioscorea Zingiberensis* (both monocotyledons), also have a conservative CYP90B for the catalyst of C22 hydroxylation of CHOL (S22H) and a bifunctional enzyme coding gene of C16 hydroxylase and C22 hydroxyl oxidase (S22O-16H) which participated in the synthesis of steroidal saponins instead of BRs respectively (Fig. 4 A B). This suggested that there is a conserved evolution of the CYP90 family gene in both monocotyledons and dicotyledons, in which these CYP90 genes may be evaluated from their common ancestor CYP90B, whose copies evolved with duplication followed by loss of function or neofunctionalization [10, 22]. The CYP72A and CYP94D were predicted as S26H in several plants. The independent sister clade of CYP72A and CYP94D clades also contained 2 copy S26H genes (08.1961 and 08.2077) with higher expression in Rs of both green and purple asparagus were detected as S26H gene in asparagus. Additionally, most of the USSP genes identified in asparagus had two or more copies, such as HMGSs, HMGRs, IDIs, C14Rs, CASs, etc., which are also consistent with the gene doubling event of SSP in asparagus. These results suggest that duplication and following variations of SSP genes are important to produce and accumulate the diversified steroidal saponins in different organs of asparagus.

It is reported that, compared with leaves and rhizomes, almost all genes related to diosgenin synthesis were highly expressed in leaves of *D. zingiberensis*, suggesting that the diosgenin may have been synthesized in leaves (source), then converted into diosgenin saponins, and finally transported to its rhizomes (sink) for storage [22]. While in this study, most DSSP genes of green and purple asparagus are highly expressed in the Rs, which is consistent with the high accumulation of saponins in Rs. However, in purple asparagus, most USSP genes for CHOL synthesis were not only up-regulated expression in Rs but also expressed mildly in Ss and Fs. It seems that the expression of DSSP genes in green and purple asparagus was relatively consistent, but had differences in the expression of CHOL synthetic (USSP) genes between asparagus cultivars. These findings suggest that steroidal synthesis and regulation genes expression are specific in different species, even though the synthetic pathway in both asparagus and *D. zingiberensis* are similar.

In this study, 18 steroidal metabolites were detected, suggesting a diversified pathway for various steroidal saponins synthesis and accumulation from CHOL. We propose a complete SSP with regulatory networks in asparagus. Although the predicted genes in SSP need to be further functionalized by molecular biology, the proposed pathway provides a way to synthesize the active saponins compounds of asparagus with the strategy of synthetic biology.

It is generally believed that glycosylation is the major step of steroidal saponins synthesis, and the modified sugar groups play a crucial role in increasing solubility, changing the chemical properties and pharmacological activities of steroidal saponins. A *SaGT4A* gene cloned from *Solanum aculeatissimum* involved in the steroidal synthesis plays a role in the plant defense system. The *SaGT4A* was identified as the first steroid 3-O-glycosyltransferase (S3βGT) [23]. Thereafter, S3βGTs in potatoes and other plants were also identified with homologous searching [24]. In this study, 18 GTs were detected as up-regulated genes in Rs, 3 of them (*04.386*, *04.381* and *10.1805*) with 2 genes duplicated were highly homologous with the characterized S3βGTs with the conserved amino acid motifs (Fig. 4E), the detected S3βGTs in asparagus are likely to participate in glycosylation modification of C3-OH group in steroidal aglycones. Considering 18 kinds of steroidal saponins in the three types of steroidal aglycones, these GTs up-regulated in Rs may not only be involved in the glycosylation of furostanol saponins of C3-glycosyl groups but also play roles in improving stress tolerance. Therefore, these GT genes in asparagus may also be used as stress tolerance-improving genes for both asparagus and other crops' stress tolerance improvements with method of genetic engineering.

In this study, 22R-OH furostanol (22R-OH-Fu) is a key intermediate for the synthesis of steroidal saponins, and the 26-O-glycosyltransferase (F26GT) or the 26-O-β-glucosidase (F26G) may have also participated in glycosyl modification and removal of C26-O sugar group to determine whether spirostanol/isospirostanol steroids or furostanol saponins are synthesized. Based on chemical structure, furostanol saponins (e.g. protodioscin) resulted from glycosylation modification at C3-OH and/or C26-OH of 22R-OH-Fu with the opening of its F ring; while spirostanol/isospirostanol saponins with F-loop closure of steroidal skeleton also need C3-OH-glycosyl-modification by S3βGTs. According to the type and abundance of detected saponins (the furostanol saponins is lower than spirostanols/isospirostanols types of steroidal saponins), furostanol saponins are proposals as side products of SSP in our detected asparagus cutivar. When plants encounter stress, they may initiate more spirostanols/isospirostanols type steroidal saponins synthesis by hydrolyzing the 26-O-glycoside bond through F26G and rapidly produce F ring closing steroidal saponins to adapt to the stresses in organs of asparagus.

Diosgenin is an important raw material for various steroidal drugs. Currently, large-scale production of diosgenin employing synthetic biology or chemical synthesis remains costly, while direct extraction of steroidal saponins from plant resources is relatively safe and currently the main application method. However, due to the long growth years of medicinal plants and low saponin content, they do not meet the current demands [25].

The study of saponins synthetic and regulatory pathway will not only help to understand steroids' roles in plant growth and development under stress but also be of great significance to the synthesis of steroidal drugs with increasing demands with integrating methods of gene knockout of supervising genes and the overexpression of some key-step synthetic genes to optimize diosgenin synthesis in microorganisms or plants as well.

## Conclusion

In this study, 18 steroidal compounds were detected by metabolomics in asparagus roots, spears and flowering twigs, with higher accumulation in roots. Joint transcriptome analyses and other bioinformatics methods screened 16 TFs and 45 structural (synthetic) genes. The key genes in DSSP focused in this study include; S22H, S16H, S22O-16H, S26H, S3βGT and F26GHs. The optimized annotations of all screened genes were performed by Isoform sequencing, Furthermore, the full SSP of asparagus was proposed.

## Materials and methods

### Plant material

The roots (Rs), spears (Ss) and flowering twigs (Fs) of both the green Asparagus cultivar "Gurlph Millennium" (G) and the purple Asparagus cultivar "Purple Passion" (P) planted in the field of Yunnan Agricultural University, named as GRs, GSs, GFs, PRs, PSs and PFs (Fig. 1A-F) respectively, were sampled with 3 biological replications. The samples, which were stored at -80 ˚C, were used for the analysis of metabolites using UPLC -MS, and gene expression detection using methods of both Illumina RNAseq and PacBio IsoSeq.

### Illumina RNAseq and data processing

The RNAs were extracted from Rs, Ss and Fs of green and purple asparagus for RNAseq using Illumina platform, and clean data was obtained by quality control of raw data with FastQC [26] followed by Fastp (https://github.com/OpenGene/fastp) processed with default parameters. Clean data was mapped to *A. officinalis* reference genome downloaded from Phytozome (https://phytozome-next.jgi.doe.gov/) using Hisat2 [27], and expression quantification was performed using scripts of Featurecounts R [28] to obtain the matrix of counts, TPM (normalizing expression unit of transcripts per kilobase of exon per million mapped fragments) and FPKM (normalizing expression unit of fragments per kilobase of exon per million mapped fragments). All gene expression analyses were based on FPKM matrix, while the differential gene expression was analyzed by Bioconductor packages of DEseq2 [29] with the counts matrix. The predicted protein sequences of the asparagus genome were submitted to eggNOG (http://eggnog-mapper.embl.de/) for annotation. The expression of FPKM matrix of 18 asparagus samples was filtered by median absolute deviation (MAD) and used to perform co-expression analysis by R package of WGCNA for co-expressed gene modules [30]. The abundance of both steroidal and terpenoid metabolites were associated with gene modules obtained from WGCNA analysis. The saponins synthesized related gene modules were selected with critical standards of absolute correlation values ≥ 0.7 and P-value ≤ 0.05 using the spearman method. Principal components analysis (PCA) was performed using the expression matrix (FPKM) of all genes and abundance of steroid and terpenoid metabolites by R package PCAtools [31], respectively. GO and KEGG enrichment of differential expression genes (DEGs) or interested genes correlated to steroid contents in obtained gene modules by WGCNA analysis were performed using R package of ClusterProfiler [32].

### Pacbio IsoSeq and data processing

For optimizing genome annotations and detection of the possible alternative exon splicing and gene fusion of synthetic and regulatory-related genes of SSP, the full-length transcripts were detected with the mixture of RNAs extracted from samples of Rs, Ss, and Fs of purple passion male, female and andromonoecious respectively. The obtained Pacbio data were analyzed by IsoSeq3 pipelines (https://github.com/ ErisonChen/IsoSeq), and the polished CCS subreads were generated from the subreads bam files by CCS (https://github.com/ PacificBiosciences/ccs) with a minimum quality of 0.9. The default minimum number of fullsubreads (FLs) (*n* = 3) required to generate CCS for a zero-mode waveguide (ZMW) was used. FL transcripts were determined when the sequences had the poly(A), the 5′ and 3′ cDNA primers. Lima (https://github.com/fluffos/lima) and IsoSeq3 were used to remove the primers and poly(A) tails, respectively. The clustering algorithm of ICE was used to obtain high-quality FL consensus sequences. The consensus transcripts were mapped to the *A. officinalis* reference genome using minimap2 [33] and desalt [34] respectively. The obtained BAM files were sorted and used to collapse redundant isoforms using Cupcake (https://github.com/Magdoll/ cDNA_Cupcake). The unmapped and poorly mapped isoforms were used as input to code genome reconstruction tool Cogent (https://github.com/Magdoll/ Cogent) to reconstruct the fake genome. The reconstructed fake genome was used for processing and collapsing through the ToFU IsoSeq3 pipeline to get qualified long-read transcriptome data. The *A. officinalis* reference genome sequence with its annotated gene transfer format (GTF) file and quantified long-read transcriptome data were used as input in SQANTI2 [35] to characterize/classify the collapsed isoforms and assess the quality of the sequencing data [36]. Transcript isoforms are defined as partially overlapping with exons/introns of an annotated gene, whereas fusion transcripts span two annotated gene loci, and artifacts were removed using SQANTI machine learning classifier. The GeneMarkS-T was implemented to predict ORFs from the corrected transcripts [37]. AStalavista5 was used to identify and classify alternative splicing (AS) events and the frequencies of the AS events including intron retaining (IR), exon skipping (ES), and alternative 3′ splicing (APAS). The transcripts which were lacking ORFs or harboring ORFs with less than 50 amino acids were aligned to all miRNA databases of miRbase6 (release 22.1) and Rfam database to identify possible pre-miRNAs and other non-coding RNA. The remaining not matched transcripts, which are more than 200 bp, were assessed for coding potential using CPC (CPC score ≤ 1) [38]. The resulting transcripts were considered putative lncRNA transcripts.

### Detection of metabolic compounds

The fresh Rs, Ss and Fs of asparagus were freeze-dried using a lyophilizer (Scientz-100F, Ningbo, China) and crushed to power. 100 mg powder was extracted overnight with 1 mL of 70% methanol at 4 ℃, followed by centrifugation at 10,000 g for 10 min to get supernatant. The supernatant, which was filtered with a microporous membrane (0.22 μm pore size), was used for analysis with a UPLC-ESI–MS/MS system (UPLC, Shim-pack UFLC SHIMADZU CBM30A system, www.shimadzu.com.cn/; MS, Applied Biosystems 6500 Q TRAP, www.appliedbio systems.com.cn/). The analytical conditions were used as follows, UPLC column; Waters ACQUITY UPLC HSS T3 C18 (1.8 µm, 2.1 mm*100 mm); the mobile phase, consisting of solvent A (pure water with 0.04% acetic acid) and solvent B (acetonitrile with 0.04% acetic acid). Sample measurements were performed with an elution of linear gradient program starting from 95% A, 5% B to 5% A, 95% B within 10 min, then a composition of 5% A, 95% B was kept for 1 min. Subsequently, a composition of 95% A, and 5.0% B was adjusted within 0.10 min and kept for 5 min. The column was incubated at 40 °C with an injection volume of 2 μl. The integrated and corrected chromatographic peaks of each metabolite detected from different samples were corrected with MultiaQuant version 3.0.3 (Sciex, Darmstadt, Germany) to ensure the accuracy of analyses. Based on the MetWare metabolism self-built plant DataBase (MWDB) (https://cloud.metware.cn/), the qualitative analysis of substances was carried out according to the secondary mass spectrometry information after removing the isotopic and repeated signals of K^+^, Na^+^, and NH_4_^+^ ions. The multiple reaction monitoring (MRM) mode of a triple quadrupole mass spectrometry quantified the metabolites [39] using Analyst 1.6.3 (AB Sciex) with default parameters. Quality control samples were prepared by mixing sample extracts and analyzing the repeatability treated by the same methods. Significantly different metabolites between groups were determined by R packages of MetaboAnalystR (https://github.com/ xia-lab/MetaboAnalystR/) and ropls (http://www.bioconductor.org/packages/ release/bioc/html/ropls. html), selected with standard critical VIP ≥ 1 and absolute Log2FC (fold change) ≥ 1.

### Determination of total saponins contents

The total steroidal saponins contents were assayed with the Spectrophotometric method [40] with modifications by using 3 biological replicates of Rs, Ss, and Fs of both green(G) and purple Asparagus(P). The diosgenin was used as the standard substance to obtain the linear regression equation of y = 0.008x-0.023 with R^2^ = 0.9946 (y: relative content of saponins, x: absorbance_457nm_ of resection mixture diosgenin). About 0.25 g of fresh sample was grounded to power with liquid nitrogen, followed by adding 10 mL of 95% ethanol to sonicate for 30 min into a homogenate, then diluting to get a final mixture of 50 mL with 75% ethanol. Then centrifuged at 10,000 rpm for 10 min to obtain supernatant. 0.3 mL of the extracted solution was evaporated to dry residues at 60 °C, then 0.2 mL of 5% vanillin in acetate and 0.8 mL of perchloric acid were added, mixed and incubated at 60 °C for 15 min. After cooling on ice for 5 min, 10 mL of acetate was added to develop color for 20 min at room temperature. The absorption at 457 nm was measured with a UV–Vis to calculate the content of total steroidal saponins.

### Genes expression analysis with qRT-PCR assay

The RNAs of the Rs, Ss, and Fs of purple asparagus were extracted using the RNA Easy Fast Plant Tissue Kit, reverse transcriptions were conducted to get cDNA using the Fasting RT Kit, and qRT-PCR were analyzed using SuperReal PreMix Plus kit with SYBR Green respectively. All kits of the above were provided by Tiangen Biotech (Beijing, China). The PCR primers, which were designed based on the annotated genome of asparagus, were chemically synthesized from Shenggong Biotech (Shanghai, China), listed in Table S3.

## Supplementary Information

**Additional file 1:** **Table S1.** The Summary of RNASeq. **Table S2.** The summary of Iso-Seq. **Table S3.** Primers of qRT-PCR. **Table S4.** The prediction of steroids-related TFs. **Table S5.** Summary of RNA-seq and ISO-seq analysis of steroid-related genes and TFs.

**Additional file 2:** **Fig. S1.** The structures and names of steroid and terpenoid metabolites. The compounds numbered C01~C20 were detected in green and purple asparagus, while the compounds without No. existed in theory. Each box means a type of saponin with a similar structure,  whose aglycones were marked with red. The green box indicates the full name of the sugar groups. **Fig. S2.** Transcriptome and metabolome analyses. A, the PCA of all metabolites detected in green and purple asparagus. B, C and D, the KEGG enrichment analyses among GRs vs PRs, GSs vs PSs and GFs vs PFs, separately. E, the venn diagrams of differential metabolites (DMs, the left) and differentially expressed genes (DEGs, the right) in GRs vs PRs, GSs vs PSs and GFs vs PFs; F and G, the venn diagrams of DMs and DEGs in GRs vs GSs, GRs vs GFs, PRs vs PSs, PRs vs PFs respectively. **Fig. S3.** The dendrogram and correlation heatmap of the co-expression gene module. The modules highlighted with red and blue in the dendrogram represent the positive- and negative-correlated modules with steroid metabolites, respectively. **Fig. S4.** The clustering trees of cholesterol synthesis genes. The clustering tree is made up of numerous clades, in which each clade is denoted by a different color representing the same cholesterol synthetic gene derived from different organisms. The cholesterol synthesis genes of the other organisms are named concerning their protein symbols followed by NCBI access No. then the organism name, while that of asparagus is called using their respective genomic protein IDs. **Fig. S5.** The phylogenetic tree of CY450s superfamilies in asparagus(AoCYP450)related to DSSP. The tree was constructed using selected AoCYP450 genes and functionally characterized CYP450 family genes, including CYP90B, CYP90GandCYP94 and CYP72, based on protein sequence similarity with MEGAX by NJ. **Fig. S6.** The multiple sequence alignment (MSA) of 03.2646, 03.698, BR- and saponin-related CYP90Bs was performed with Jalview. The darker the color, the more conservative Aa resitues is. The differentially conserved amino acid residues are highlighted in red. **Fig. S7.** The MSA of 03.2424 and CYP90Gs that functionally characterized as sterol C16S hydroxylase and C22-keto oxidase. The darker the color, the more conservative base is. The important conserved amino acid residues among sequences were highlighted in red. **Fig. S8.** Identification of C16 hydroxylase in asparagus. The tree was constructed using TBtools with protein motifs predicted by MMEM, and the MSA was constructed with 16DOXs of potato and tomato using Jalviwe. The key domains were heightened in red. **Fig. S9.** The cis-active elements prediction of all steroid-related genes’ and TFs’ promoters was performed using TBtools. **Fig. S10.** The chromosome location and fusions genes of SSP detected with Pacbio Isoseq data. The fused genes were highlighted in red linker with the blueids.

**Additional file 3.** 

## Data Availability

The datasets generated during the current study are available in the National Genomics Data Center repository with No GSA CRA009175, https://ngdc.cncb.ac.cn/.

## Abbreviations

SSP: The steroidal saponins biosynthesis pathway
USSP: The upstream biosynthetic pathway of steroidal saponins
DSSP: The downstream biosynthetic pathway of steroidal saponins
FPKM: Normalizing expression unit of fragments per kilobase of exon per million mapped fragments
CHOL: Cholesterol
22-OH-CHOL: 22-Hydroxycholesterol
BRs: Brassinosteroids
S22H: Steroid-22-hydroxylase
S22-O-16-H: Steroids-22-oxidase-16-hydroxyulase
S26H: Steroids-26-hydroxyulase
3-GT: Steroids-3-β-glycosyltransferase
F26GH: Furostanol glycoside 26-O-beta-glucosidases
DEGs: Differential expression genes
UDEGs: Up-regulated differentially expressed genes
DMs: Differential metabolites
UDMs: Up-regulated differential metabolites
