# Supplementary material for: Steroidal saponin profiles and their key genes for synthesis and regulation in Asparagus officinalis L. by joint analysis of metabolomics and transcriptomics
Source: BMC Plant Biol. 2023 Apr 20;23:207. doi: 10.1186/s12870-023-04222-x (PMC10116787; doi:10.1186/s12870-023-04222-x)
Supplement: Supplementary file 1 — Additional file 1: Table S1. The Summary of RNASeq. Table S2. The summary of Iso-Seq. Table S3. Primers of qRT-PCR. Table S4. The prediction of steroids-related TFs. Table S5. Summary of RNA-seq and ISO-seq analysis of steroid-related genes and TFs. [file 12870_2023_4222_MOESM1_ESM.docx]

| **Table S1. The Summary of RNASeq** | | | | | |
| --- | --- | --- | --- | --- | --- |
| **sample** | **Total reads** | **total bases** | **average length** | **average quality** | **percentage of properly paired reads (%)** |
| GR_1 | 45043178 | 6756476700 | 150 | 36.10 | 77.10 |
| GR_2 | 43465016 | 6519752400 | 150 | 36.10 | 77.80 |
| GR_3 | 60475348 | 9071302200 | 150 | 36.10 | 76.80 |
| GS_1 | 48224874 | 7233731100 | 150 | 36.10 | 82.00 |
| GS_2 | 46305834 | 6945875100 | 150 | 36.10 | 82.00 |
| GS_3 | 51598854 | 7739828100 | 150 | 36.10 | 82.00 |
| GF_1 | 51415710 | 7712356500 | 150 | 36.10 | 81.80 |
| GF_2 | 61836306 | 9275445900 | 150 | 36.10 | 82.30 |
| GF_3 | 54692300 | 8203845000 | 150 | 36.10 | 81.90 |
| PR_1 | 84457944 | 12644401538 | 149 | 35.70 | 61.30 |
| PR_2 | 83663350 | 12520587540 | 149 | 35.80 | 60.70 |
| PR_3 | 84814446 | 12693196834 | 149 | 35.80 | 59.20 |
| PS_1 | 94526776 | 14134846962 | 149 | 35.80 | 74.10 |
| PS_2 | 83952364 | 12548679742 | 149 | 35.70 | 71.70 |
| PS_3 | 95884308 | 14326070388 | 149 | 35.70 | 73.40 |
| PF_1 | 101233094 | 15129523882 | 149 | 35.80 | 70.40 |
| PF_2 | 110929796 | 16568601506 | 149 | 35.80 | 71.30 |
| PF_3 | 80426088 | 12017530292 | 149 | 35.80 | 70.90 |
| Total | 1282945586 | 192042051684 | - | 35.93 | 74.26 |

| **Table S2. The summary of Iso-Seq** | | | | | |
| --- | --- | --- | --- | --- | --- |
| **Sample** | **CCS Number** | **Number of consensus isoforms** | **Average consensus isoforms read length** | **Number of full-length non-chimeric reads** | **Full-length non-chimeric percentage (FLNC%)** |
| F01 | 684004 | 60475 | 2223 | 586653 | 85.77 |
| F02 | 330570 | 132567 | 2159 | 248828 | 75.27 |
| F03 | 592029 | 234367 | 1817 | 435941 | 73.64 |

| **Table S3. Primers of qRT-PCR** | | | |
| --- | --- | --- | --- |
| **gene_id** | **Primer name** | **Sequence** |  |
| evm.model.AsparagusV1_05.1543 | 05.1543_F | CCAGTCAGCGTGCTTGGA |  |
|  | 05.1543_R | CACCAGCCAGGACTGAGC |  |
| evm.model.AsparagusV1_08.1961 | 08.1961_F | AACGCGAAGGAAGCCGAA |  |
|  | 08.1961_R | ACCCTCGGTGCAATGGTG |  |
| evm.model.AsparagusV1_08.1158 | 08.1158_F | GCGATTGCGGAACCCTCT |  |
|  | 08.1158_R | ACTCCTCACACTGTGCGC |  |
| evm.model.AsparagusV1_08.2077 | 08.2077_F | AACGCGAAGGAAGCCGAA |  |
|  | 08.2077_R | CGTGCAAGTGAGGCAGGA |  |
| evm.model.AsparagusV1_08.1302 | 08.1302_F | CTCATCCTCCGACGACGC |  |
|  | 08.1302_R | CACAGGCATGACACGGGT |  |
| evm.model.AsparagusV1_07.13 | 07.13_F | AAGGAAGCTGCAGCCAGG |  |
|  | 07.13_R | CTGGTGCCTAGCCATCGG |  |
| evm.model.AsparagusV1_04.76 | 04.76_F | GGCCATGGACCGAGGATG |  |
|  | 04.76_R | CCCACACCGTAGAAGCCC |  |
| evm.model.AsparagusV1_04.386 | 04.386_F | GCCGTGGTTGGGGAGATC |  |
|  | 04.386_R | TGTGCCAGCACCTCCATG |  |
| evm.model.AsparagusV1_04.2945 | 04.2945_F | TGGTTGGTCAGCGGATCG |  |
|  | 04.2945_R | GTGAAGGGATGCCGACCA |  |
| evm.model.AsparagusV1_04.1653 | 04.1653_F | GAGCATCCACGTCGAGGG |  |
|  | 04.1653_R | ACTGGAGGTGCCGTTGTC |  |
| evm.model.AsparagusV1_03.698 | 03.698_F | AATGTCGTTCAGCGGGGG |  |
|  | 03.698_R | GGATAGTCGTGCTCGGCC |  |
| evm.model.AsparagusV1_03.2424 | 03.2424_F | CCGACGATCATCTCCGCC |  |
|  | 03.2424_R | TCCCGAGGATGTCAGCCA |  |
| evm.model.AsparagusV1_09.871 | 09.871_F | AAATCCCGCTGGAGTGGC |  |
|  | 09.871_R | TCTCCAAAGCCAGCGGAG |  |
| evm.model.AsparagusV1_09.1129 | 09.1129_F | TGGGAGAGGACCGAGCAT |  |
|  | 09.1129_R | GAAGGCCACTCCAGCTGG |  |

| **Table S4. The prediction of steroids-related TFs** | | | |
| --- | --- | --- | --- |
| **gene_id** | **TF family** | **Corresponding TFs in *Arabidopsis thaliana*** | **description** |
| evm.model.AsparagusV1_02.664 | NAC | AT1G01720.1 | NAC family protein |
| evm.model.AsparagusV1_01.2664 | TALE | AT1G23380.1 | KNOTTED1-like homeobox gene 6 |
| evm.model.AsparagusV1_08.1302 | C2H2 | AT1G27730.1 | salt tolerance zinc finger |
| evm.model.AsparagusV1_07.2491 | bHLH | AT1G29950.1 | bHLH family protein |
| evm.model.AsparagusV1_08.142 | HSF | AT1G32330.1 | heat shock transcription factor A1D |
| evm.model.AsparagusV1_01.1829 | C3H | AT1G32360.1 | C3H family protein |
| evm.model.AsparagusV1_08.185 | NAC | AT1G33060.2 | NAC 14 |
| evm.model.AsparagusV1_07.1206 | MYB | AT1G48000.1 | myb domain protein 112 |
| evm.model.AsparagusV1_05.2213 | Dof | AT1G51700.1 | DOF zinc finger protein 1 |
| evm.model.AsparagusV1_02.591 | ARF | AT1G59750.2 | auxin response factor 1 |
| evm.model.AsparagusV1_08.3285 | NAC | AT1G77450.1 | NAC domain containing protein 32 |
| evm.model.AsparagusV1_03.2814 | TALE | AT2G16400.1 | BEL1-like homeodomain 7 |
| evm.model.AsparagusV1_09.607 | bHLH | AT2G22760.1 | bHLH family protein |
| evm.model.AsparagusV1_09.609 | bHLH | AT2G22760.1 | bHLH family protein |
| evm.model.AsparagusV1_Unassigned.1070 | bHLH | AT2G22760.1 | bHLH family protein |
| evm.model.AsparagusV1_01.2373 | HSF | AT2G26150.1 | heat shock transcription factor A2 |
| evm.model.AsparagusV1_08.2354 | ERF | AT2G33710.1 | ERF family protein |
| evm.model.AsparagusV1_05.2671 | MYB | AT2G36890.1 | MYB family protein |
| evm.model.AsparagusV1_05.330 | GRAS | AT2G37650.1 | GRAS family protein |
| evm.model.AsparagusV1_07.13 | MYB | AT2G38090.1 | MYB family protein |
| evm.model.AsparagusV1_01.1427 | G2-like | AT2G38300.1 | G2-like family protein |
| evm.model.AsparagusV1_04.1653 | WRKY | AT2G38470.1 | WRKY DNA-binding protein 33 |
| evm.model.AsparagusV1_Unassigned.1052 | SBP | AT2G42200.1 | squamosa promoter binding protein-like 9 |
| evm.model.AsparagusV1_05.2715 | bZIP | AT2G46270.1 | G-box binding factor 3 |
| evm.model.AsparagusV1_04.714 | MYB | AT3G11280.1 | MYB family protein |
| evm.model.AsparagusV1_05.2823 | C3H | AT3G12680.1 | floral homeotic protein (HUA1) |
| evm.model.AsparagusV1_01.3001 | NAC | AT3G18400.1 | NAC domain containing protein 58 |
| evm.model.AsparagusV1_03.2136 | bHLH | AT3G19500.1 | bHLH family protein |
| evm.model.AsparagusV1_04.76 | MYB | AT3G23250.1 | myb domain protein 15 |
| evm.model.AsparagusV1_03.79 | bHLH | AT3G47640.2 | bHLH family protein |
| evm.model.AsparagusV1_08.1158 | C2H2 | AT3G50700.1 | indeterminate(ID)-domain 2 |
| evm.model.AsparagusV1_Unassigned.595 | ERF | AT3G54320.2 | ERF family protein |
| evm.model.AsparagusV1_02.380 | WRKY | AT3G56400.1 | WRKY DNA-binding protein 70 |
| evm.model.AsparagusV1_07.1636 | bHLH | AT3G56970.1 | bHLH family protein |
| evm.model.AsparagusV1_08.1764 | ERF | AT4G06746.1 | related to AP2 9 |
| evm.model.AsparagusV1_10.1876 | MYB | AT4G13480.1 | myb domain protein 79 |
| evm.model.AsparagusV1_Unassigned.965 | NF-YB | AT4G14540.1 | nuclear factor Y, subunit B3 |
| evm.model.AsparagusV1_01.2787 | bHLH | AT4G20970.1 | bHLH family protein |
| evm.model.AsparagusV1_06.597 | bHLH | AT4G20970.1 | bHLH family protein |
| evm.model.AsparagusV1_03.863 | MYB | AT4G21440.1 | MYB-like 102 |
| evm.model.AsparagusV1_05.613 | bHLH | AT4G30980.1 | LJRHL1-like 2 |
| evm.model.AsparagusV1_05.3567 | Trihelix | AT4G31270.1 | sequence-specific DNA binding TF |
| evm.model.AsparagusV1_05.3514 | WRKY | AT4G31550.2 | WRKY DNA-binding protein 11 |
| evm.model.AsparagusV1_05.3371 | WRKY | AT4G31800.1 | WRKY DNA-binding protein 18 |
| evm.model.AsparagusV1_08.8 | DBB | AT4G38960.1 | DBB family protein |
| evm.model.AsparagusV1_10.1203 | NAC | AT5G04410.1 | NAC domain containing protein 2 |
| evm.model.AsparagusV1_06.1857 | NF-YA | AT5G06510.2 | nuclear factor Y, subunit A10 |
| evm.model.AsparagusV1_02.1766 | NAC | AT5G08790.1 | NAC family protein |
| evm.model.AsparagusV1_08.983 | NF-YA | AT5G12840.2 | nuclear factor Y, subunit A1 |
| evm.model.AsparagusV1_08.776 | ERF | AT5G25190.1 | ERF family protein |
| evm.model.AsparagusV1_03.2764 | bHLH | AT5G43650.1 | bHLH family protein |
| evm.model.AsparagusV1_Unassigned.978 | bHLH | AT5G43650.1 | bHLH family protein |
| evm.model.AsparagusV1_01.3476 | HSF | AT5G45710.1 | HSF family protein |
| evm.model.AsparagusV1_04.3272 | MYB | AT5G47390.1 | MYB_related family protein |
| evm.model.AsparagusV1_03.996 | GRAS | AT5G48150.1 | GRAS family protein |
| evm.model.AsparagusV1_03.2992 | GRAS | AT5G52510.1 | SCARECROW-like 8 |
| evm.model.AsparagusV1_08.3586 | GRAS | AT5G52510.1 | SCARECROW-like 8 |
| evm.model.AsparagusV1_01.3136 | NAC | AT5G53950.1 | NAC family protein |
| evm.model.AsparagusV1_10.1562 | MYB | AT5G57620.1 | myb domain protein 36 |
| evm.model.AsparagusV1_10.1786 | C2H2 | AT5G60470.1 | C2H2 family protein |
| evm.model.AsparagusV1_08.404 | HSF | AT5G62020.1 | heat shock transcription factor B2A |
| evm.model.AsparagusV1_04.3454 | C2H2 |  |  |

| **Table S5. Summary of RNA-seq and ISO-seq analysis of steroid-related genes and TFs** | | | | | | | | |
| --- | --- | --- | --- | --- | --- | --- | --- | --- |
| **gene id** | **symbol** | **5'UTR existing** | **3'UTR existing** | **structural variation** | **Number of. APA sites** | **USSP/**  **DSSP** | **TF/**  **gene** | **If UDEGs** |
| evm.TU.AsparagusV1_01.1069 | C14R | y | y |  | 1 | up | gene | yes |
| evm.TU.AsparagusV1_01.1829 | C3H | y | y |  | 0 | - | TF | yes |
| evm.TU.AsparagusV1_01.2664 | TALE | y | y |  | 0 | - | TF | yes |
| evm.TU.AsparagusV1_01.3101 | 3bHSD | y | y | AS | 1 | up | gene | yes |
| evm.TU.AsparagusV1_01.3174 | C5-SD | y | y |  | 0 | up | gene | no |
| evm.TU.AsparagusV1_01.3552 | CAS | y | y | AS | 0 | up | gene | no |
| evm.TU.AsparagusV1_01.3575 | AACT | y | y |  | 4 | up | gene | no |
| evm.TU.AsparagusV1_01.991 | SSR2 | y | y | Fusion | 0 | up | gene | no |
| evm.TU.AsparagusV1_02.1354 | FPS | y | y |  | 1 | up | gene | no |
| evm.TU.AsparagusV1_02.380 | WRKY | y | y |  | 0 | - | TF | yes |
| evm.TU.AsparagusV1_02.908 | IDI | y | y | AS | 2 | up | gene | no |
| evm.TU.AsparagusV1_03.1030 | SS | y | y | AS | 0 | up | gene | no |
| evm.TU.AsparagusV1_03.1112 | PMVK | y | y | AS | 3 | up | gene | yes |
| evm.TU.AsparagusV1_03.2350 | SE | y | y |  | 0 | up | gene | no |
| evm.TU.AsparagusV1_03.2424 | C16,22-diO | y | y |  | 3 | down | gene | no |
| evm.TU.AsparagusV1_03.2646 | BR-CYP90B | y | y |  | 0 | down | gene | no |
| evm.TU.AsparagusV1_03.698 | C22H | y | y | Fusion | 1 | down | gene | no |
| evm.TU.AsparagusV1_03.754 | SE | y | y | AS | 0 | up | gene | no |
| evm.TU.AsparagusV1_03.863 | MYB | y | y |  | 1 | - | TF | yes |
| evm.TU.AsparagusV1_04.1135 | SMO | y | y |  | 1 | up | gene | yes |
| evm.TU.AsparagusV1_04.1317 | SMO | y | y |  | 0 | up | gene | no |
| evm.TU.AsparagusV1_04.1647 | SSR2 | y | y |  | 0 | up | gene | yes |
| evm.TU.AsparagusV1_04.1653 | WRKY | y | y |  | 3 | - | TF | yes |
| evm.TU.AsparagusV1_04.1910 | 3bHSD | y | y | AS | 3 | up | gene | no |
| evm.TU.AsparagusV1_04.2945 | HMGS | y | y | AS | 3 | up | gene | no |
| evm.TU.AsparagusV1_04.3454 | C2H2 | y | y | Fusion | 0 | - | TF | yes |
| evm.TU.AsparagusV1_04.386 | S3GT | y | y | AS | 1 | down | gene | no |
| evm.TU.AsparagusV1_04.648 | MVD | y | y |  | 0 | up | gene | no |
| evm.TU.AsparagusV1_04.76 | MYB | y | y |  | 0 | - | TF | yes |
| evm.TU.AsparagusV1_05.1543 | HMGR | y | y | Fusion | 6 | up | gene | yes |
| evm.TU.AsparagusV1_05.2213 | Dof | y | y |  | 0 | - | TF | yes |
| evm.TU.AsparagusV1_05.2383 | 7-DR | y | y | AS | 1 | up | gene | no |
| evm.TU.AsparagusV1_05.2823 | C3H | y | y |  | 3 | - | TF | yes |
| evm.TU.AsparagusV1_05.3121 | HMGR | y | y |  | 1 | up | gene | no |
| evm.TU.AsparagusV1_05.3371 | WRKY | y | y |  | 0 | - | TF | yes |
| evm.TU.AsparagusV1_05.3566 | CAS | y | y |  | 0 | up | gene | no |
| evm.TU.AsparagusV1_06.1107 | 3bHSD | n | n |  | 0 | up | gene | no |
| evm.TU.AsparagusV1_06.1175 | C5-SD | y | y |  | 2 | up | gene | yes |
| evm.TU.AsparagusV1_06.285 | CPI | y | y |  | 2 | up | gene | no |
| evm.TU.AsparagusV1_07.1206 | MYB | y | y |  | 0 | - | TF | yes |
| evm.TU.AsparagusV1_07.13 | MYB | y | y |  | 0 | - | TF | yes |
| evm.TU.AsparagusV1_07.130 | SMO | y | y | Fusion,AS | 0 | up | gene | yes |
| evm.TU.AsparagusV1_07.1595 | MVK | y | y |  | 0 | up | gene | no |
| evm.TU.AsparagusV1_07.2410 | SSR2 | y | y | Fusion,AS | 4 | up | gene | no |
| evm.TU.AsparagusV1_08.1158 | C2H2 | y | y |  | 0 | - | TF | yes |
| evm.TU.AsparagusV1_08.1190 | GGPPS | y | y |  | 0 | up | gene | no |
| evm.TU.AsparagusV1_08.1302 | C2H2 | y | y |  | 0 | - | TF | yes |
| evm.TU.AsparagusV1_08.1961 | C26H | y | y |  | 0 | down | gene | no |
| evm.TU.AsparagusV1_08.2077 | C26H | y | y |  | 0 | down | gene | no |
| evm.TU.AsparagusV1_08.3561 | SMO | y | y |  | 0 | up | gene | no |
| evm.TU.AsparagusV1_09.1129 | F26G | y | y |  | 0 | down | gene | yes |
| evm.TU.AsparagusV1_09.1161 | IDI | y | y | AS | 2 | up | gene | no |
| evm.TU.AsparagusV1_09.871 | F26G | y | y | Fusion,AS | 2 | down | gene | yes |
| evm.TU.AsparagusV1_10.1196 | HMGS | y | y | AS | 2 | up | gene | no |
| evm.TU.AsparagusV1_10.1562 | MYB | n | n |  | 0 | - | TF | yes |
| evm.TU.AsparagusV1_10.1786 | C2H2 | y | y |  | 0 | - | TF | yes |
| evm.TU.AsparagusV1_Unassigned.240 | CYP51 | y | y | AS | 9 | up | gene | yes |
| evm.TU.AsparagusV1_Unassigned.725 | C14R | y | y | AS | 1 | up | gene | yes |
| evm.TU.AsparagusV1_Unassigned.906 | F26G | y | y |  | 0 | down | gene | yes |
| evm.TU.AsparagusV1_Unassigned.932 | MVK | y | y |  | 0 | up | gene | no |
| evm.TU.AsparagusV1_Unassigned.946 | 16DOX | y | y |  | 0 | down | gene | no |

*UTR, untranslated regions. AS, alternative splicing. APA, alternative polyadenylation. USSP/DSSP, upstream/downstream biosynthetic pathway of steroidal saponins. UDEGs, up=regulated differential expression genes.
