# Supplementary material for: Steroidal saponin profiles and their key genes for synthesis and regulation in Asparagus officinalis L. by joint analysis of metabolomics and transcriptomics
Source: BMC Plant Biol. 2023 Apr 20;23:207. doi: 10.1186/s12870-023-04222-x (PMC10116787; doi:10.1186/s12870-023-04222-x)
Supplement: Supplementary file 2 — Additional file 2: Fig. S1. The structures and names of steroid and terpenoid metabolites. The compounds numbered C01~C20 were detected in green and purple asparagus, while the compounds without No. existed in theory. Each box means a type of saponin with a similar structure, whose aglycones were marked with red. The green box indicates the full name of the sugar groups. Fig. S2. Transcriptome and metabolome analyses. A, the PCA of all metabolites detected in green and purple asparagus. B, C and D, the KEGG enrichment analyses among GRs vs PRs, GSs vs PSs and GFs vs PFs, separately. E, the venn diagrams of differential metabolites (DMs, the left) and differentially expressed genes (DEGs, the right) in GRs vs PRs, GSs vs PSs and GFs vs PFs; F and G, the venn diagrams of DMs and DEGs in GRs vs GSs, GRs vs GFs, PRs vs PSs, PRs vs PFs respectively. Fig. S3. The dendrogram and correlation heatmap of the co-expression gene module. The modules highlighted with red and blue in the dendrogram represent the positive- and negative-correlated modules with steroid metabolites, respectively. Fig. S4. The clustering trees of cholesterol synthesis genes. The clustering tree is made up of numerous clades, in which each clade is denoted by a different color representing the same cholesterol synthetic gene derived from different organisms. The cholesterol synthesis genes of the other organisms are named concerning their protein symbols followed by NCBI access No. then the organism name, while that of asparagus is called using their respective genomic protein IDs. Fig. S5. The phylogenetic tree of CY450s superfamilies in asparagus(AoCYP450)related to DSSP. The tree was constructed using selected AoCYP450 genes and functionally characterized CYP450 family genes, including CYP90B, CYP90GandCYP94 and CYP72, based on protein sequence similarity with MEGAX by NJ. Fig. S6. The multiple sequence alignment (MSA) of 03.2646, 03.698, BR- and saponin-related CYP90Bs was performed with Jalview. Th [file 12870_2023_4222_MOESM2_ESM.docx]

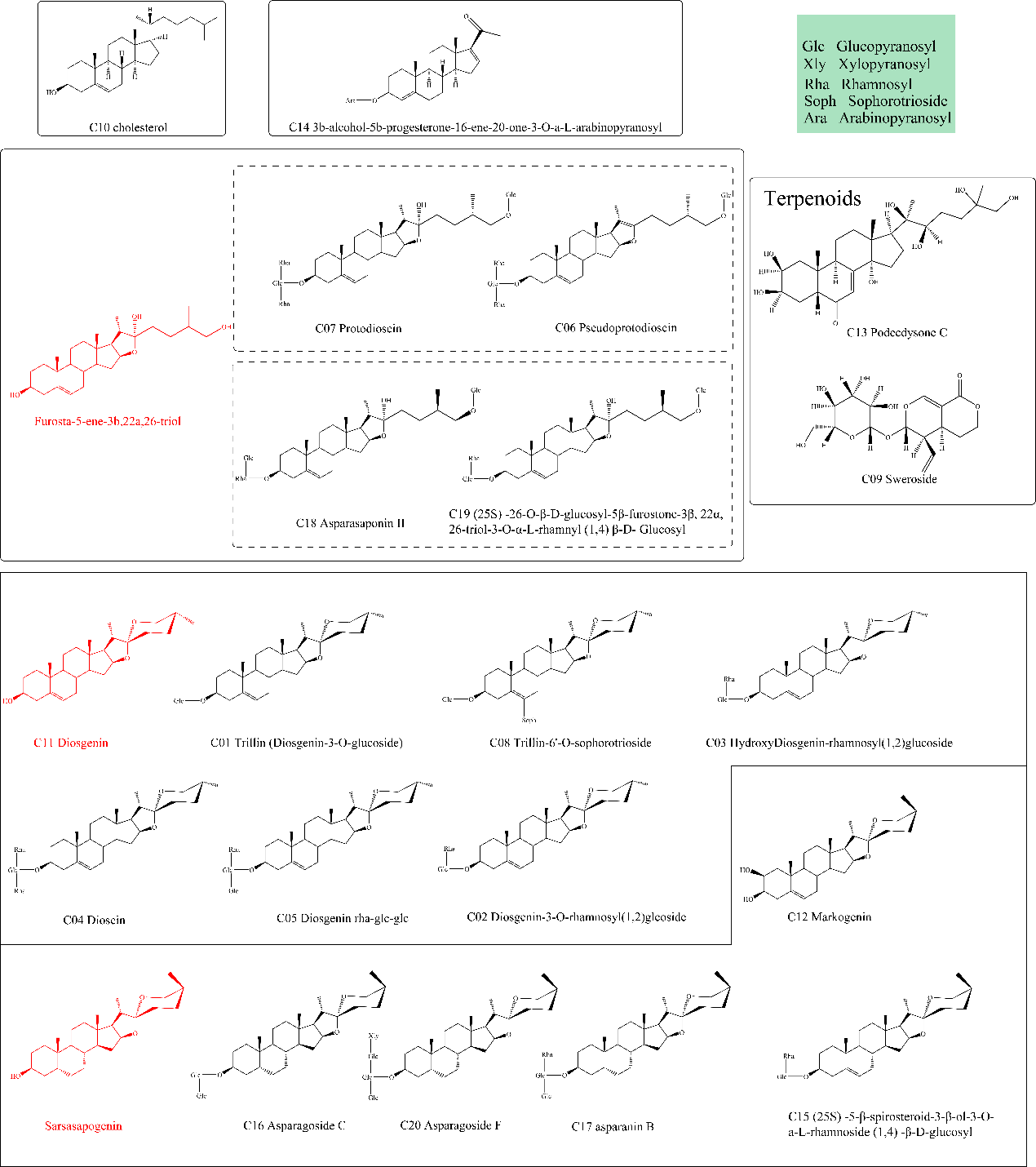


Fig S1. The structures and names of steroid and terpenoid metabolites. The compounds numbered C01~C20 were detected in green and purple asparagus, while the compounds without No. existed in theory. Each box means a type of saponin with a similar structure, whose aglycones were marked with red. The green box indicates the full name of the sugar groups.


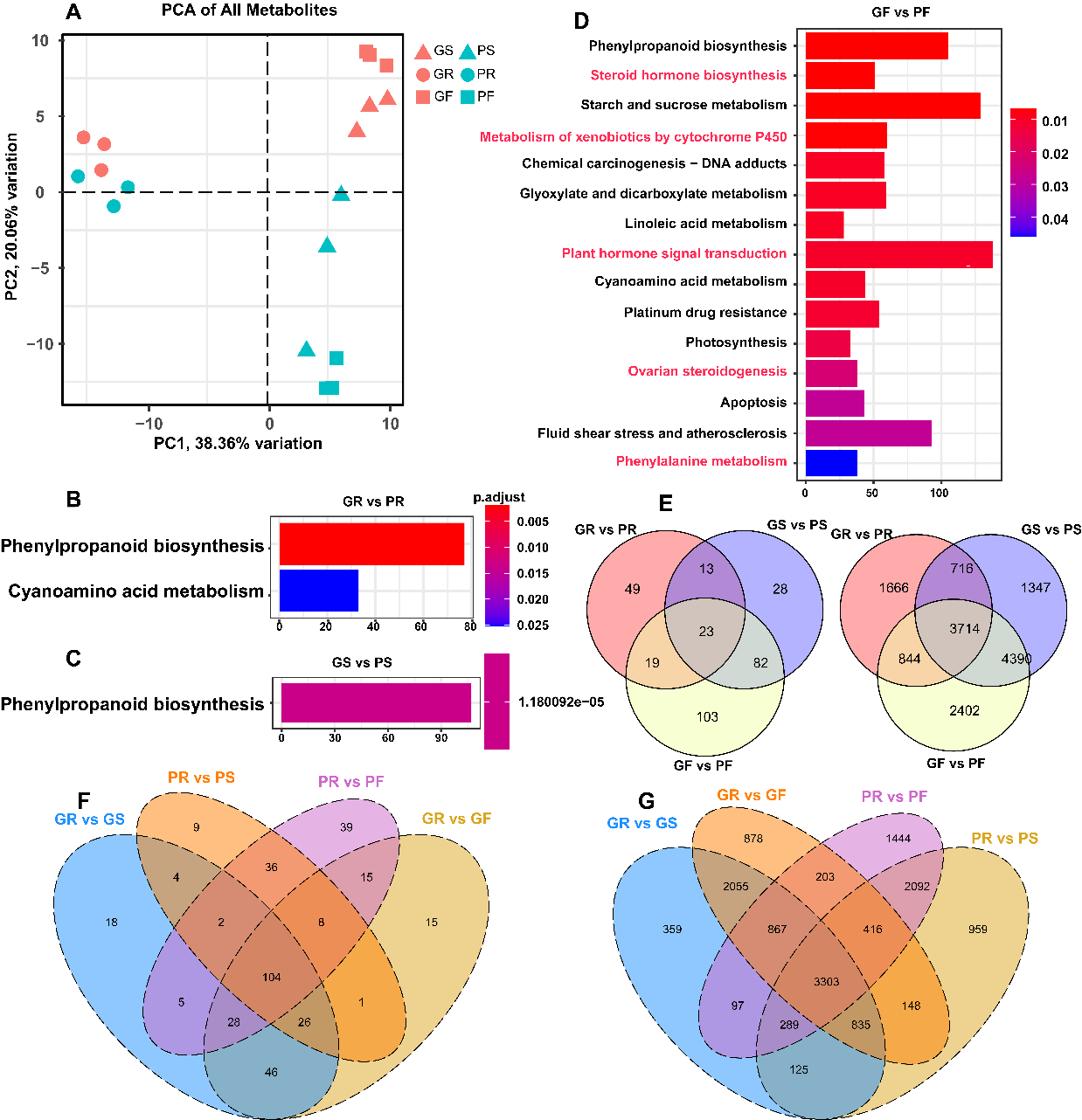


Fig S2. Transcriptome and metabolome analyses. A, the PCA of all metabolites detected in green and purple asparagus. B, C and D, the KEGG enrichment analyses among GRs vs PRs, GSs vs PSs and GFs vs PFs, separately. E, the venn diagrams of differential metabolites (DMs, the left) and differentially expressed genes (DEGs, the right) in GRs vs PRs, GSs vs PSs and GFs vs PFs; F and G, the venn diagrams of DMs and DEGs in GRs vs GSs, GRs vs GFs, PRs vs PSs, PRs vs PFs respectively.


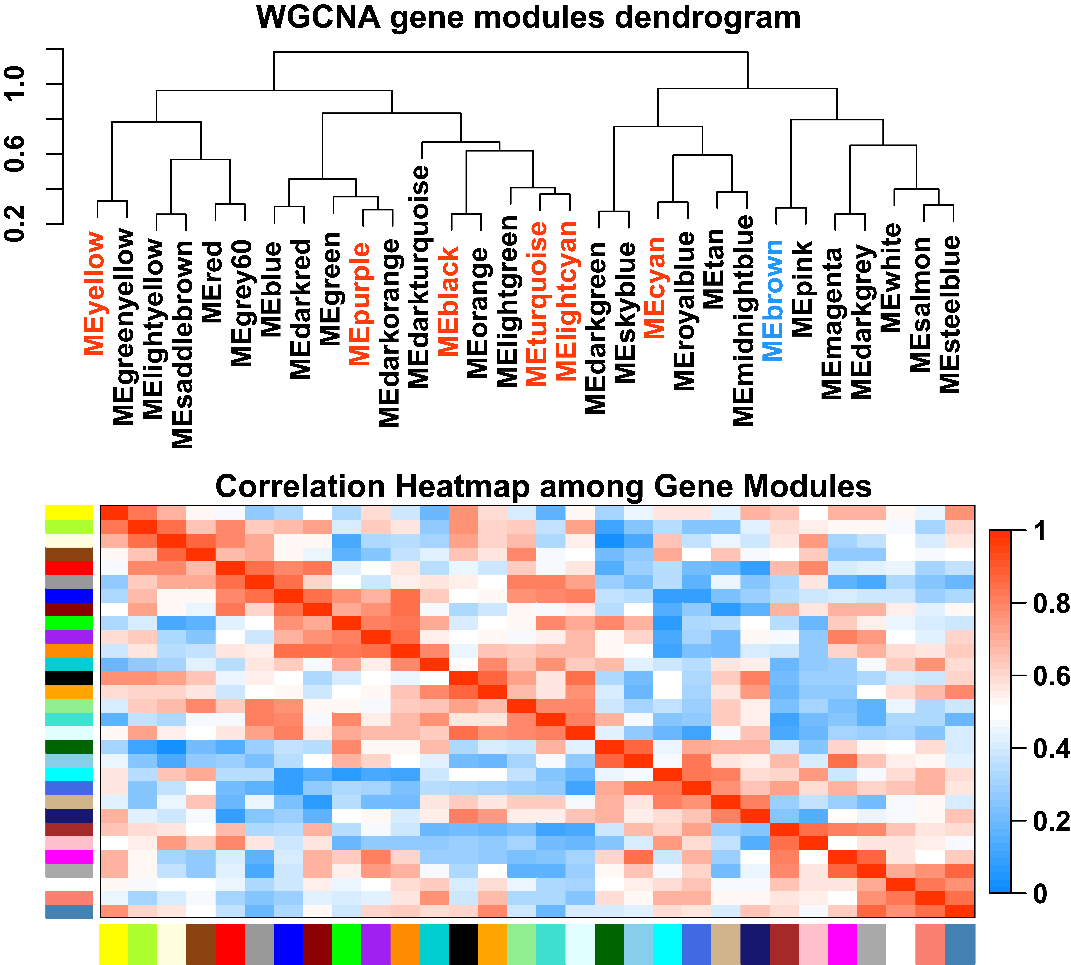


Fig S3. The dendrogram and correlation heatmap of the co-expression gene module. The modules highlighted with red and blue in the dendrogram represent the positive- and negative-correlated modules with steroid metabolites, respectively.


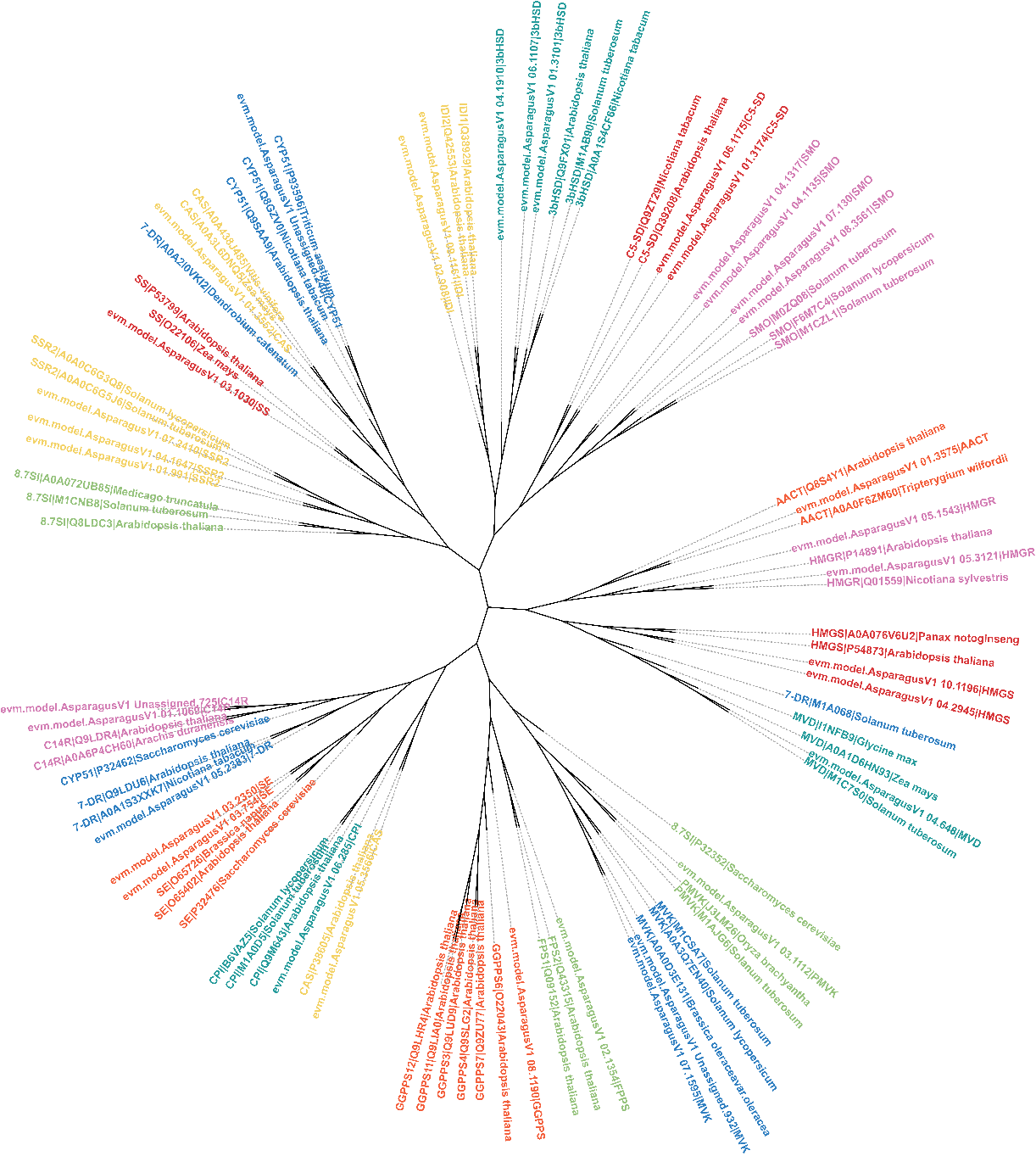


Fig S4. The clustering trees of cholesterol synthesis genes. The clustering tree is made up of numerous clades, in which each clade is denoted by a different color representing the same cholesterol synthetic gene derived from different organisms. The cholesterol synthesis genes of the other organisms are named concerning their protein symbols followed by NCBI access No. then the organism name, while that of asparagus is called using their respective genomic protein IDs.


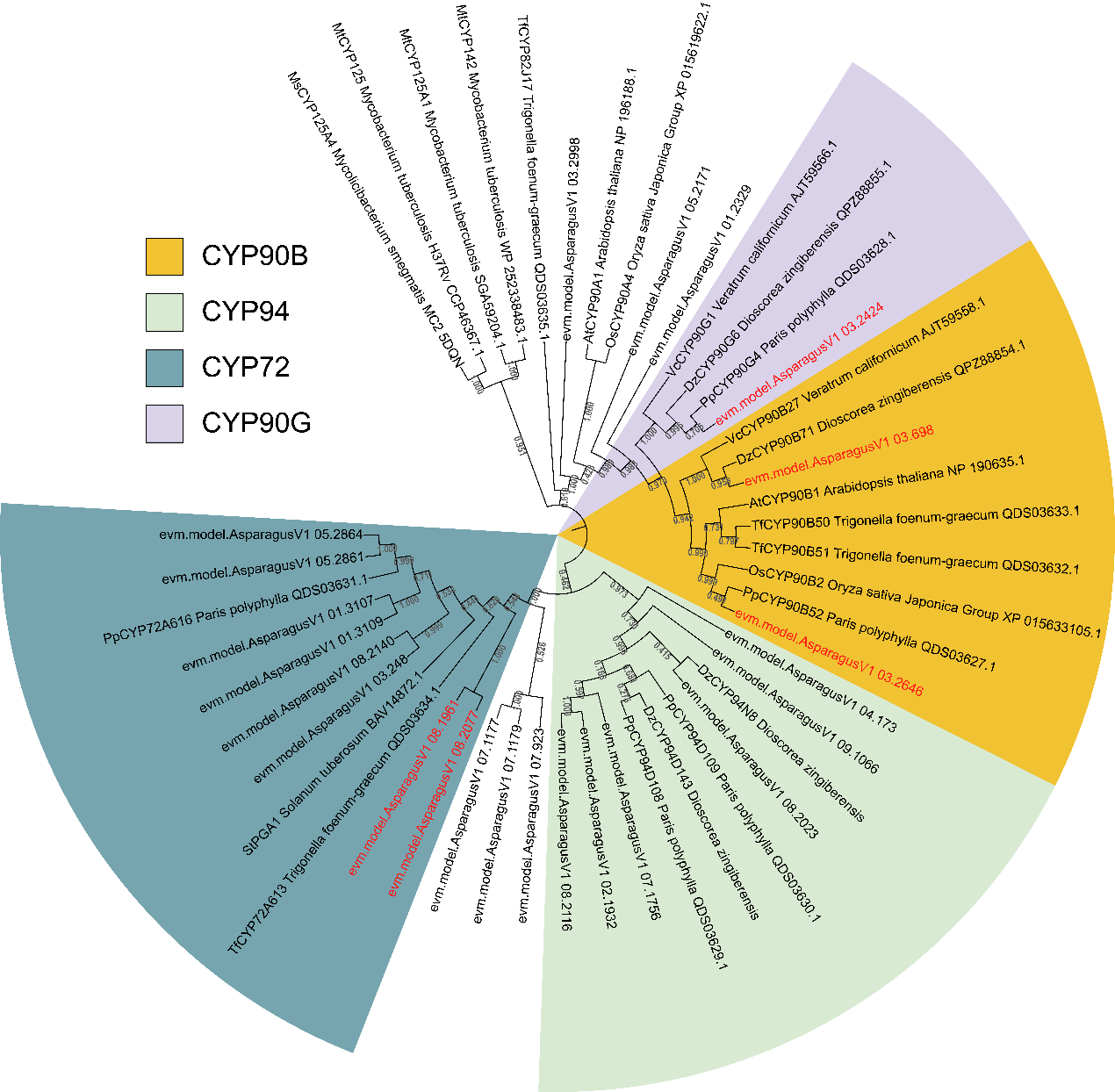


Fig S5 The phylogenetic tree of CY450s superfamilies in asparagus(AoCYP450) related to DSSP. The tree was constructed using selected AoCYP450 genes and functionally characterized CYP450 family genes, including CYP90B, CYP90G andCYP94 and CYP72, based on protein sequence similarity with MEGAX by NJ.


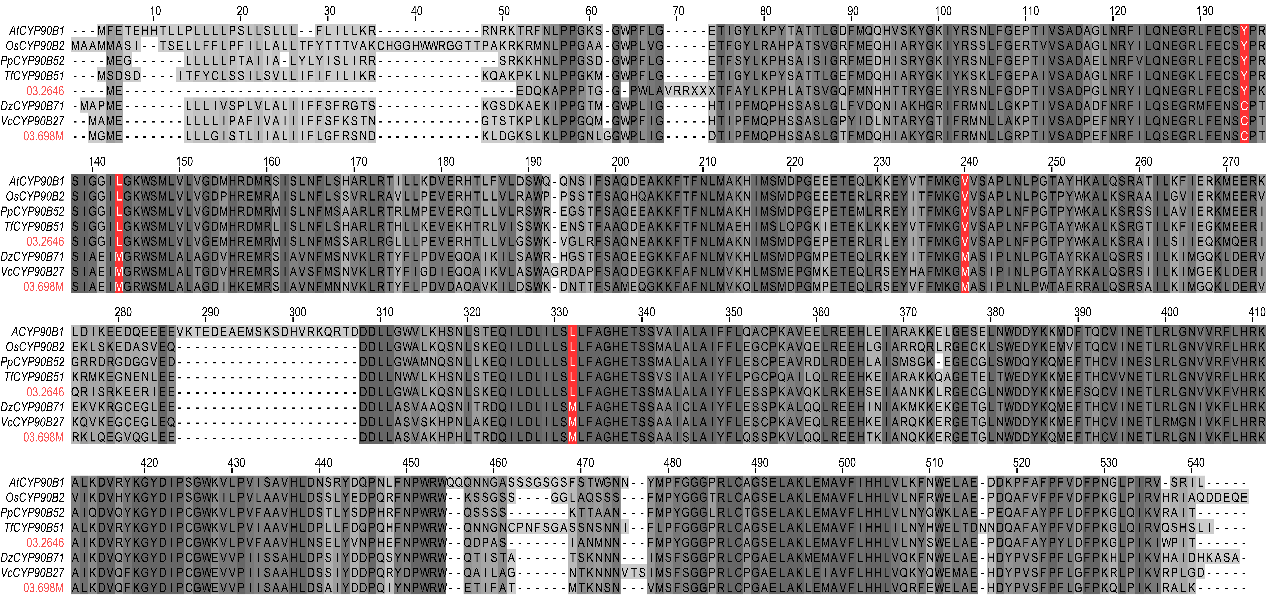


Fig S6. The multiple sequence alignment (MSA) of 03.2646, 03.698, BR- and saponin-related CYP90Bs was performed with Jalview. The darker the color, the more conservative Aa resitues is. The differentially conserved amino acid residues are highlighted in red.


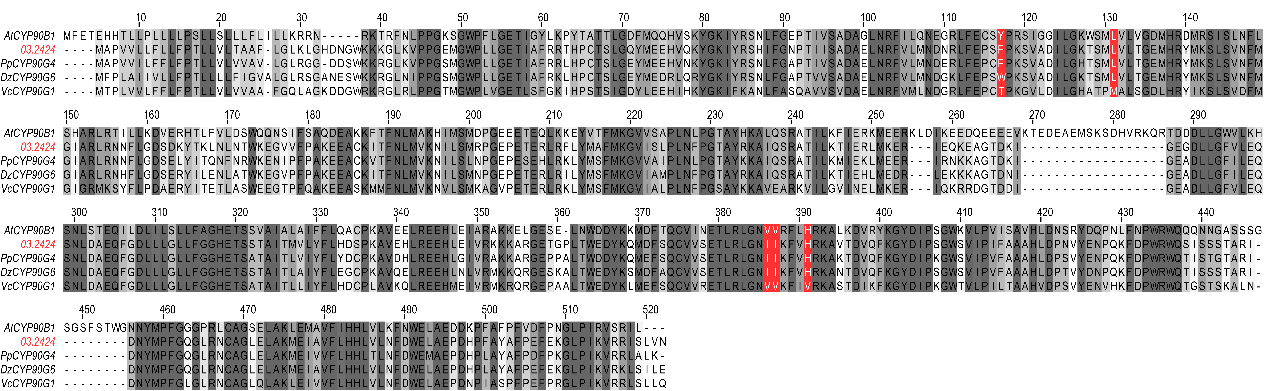


Fig S7. The MSA of 03.2424 and CYP90Gs that functionally characterized as sterol C16S hydroxylase and C22-keto oxidase. The darker the color, the more conservative base is. The important conserved amino acid residues among sequences were highlighted in red.


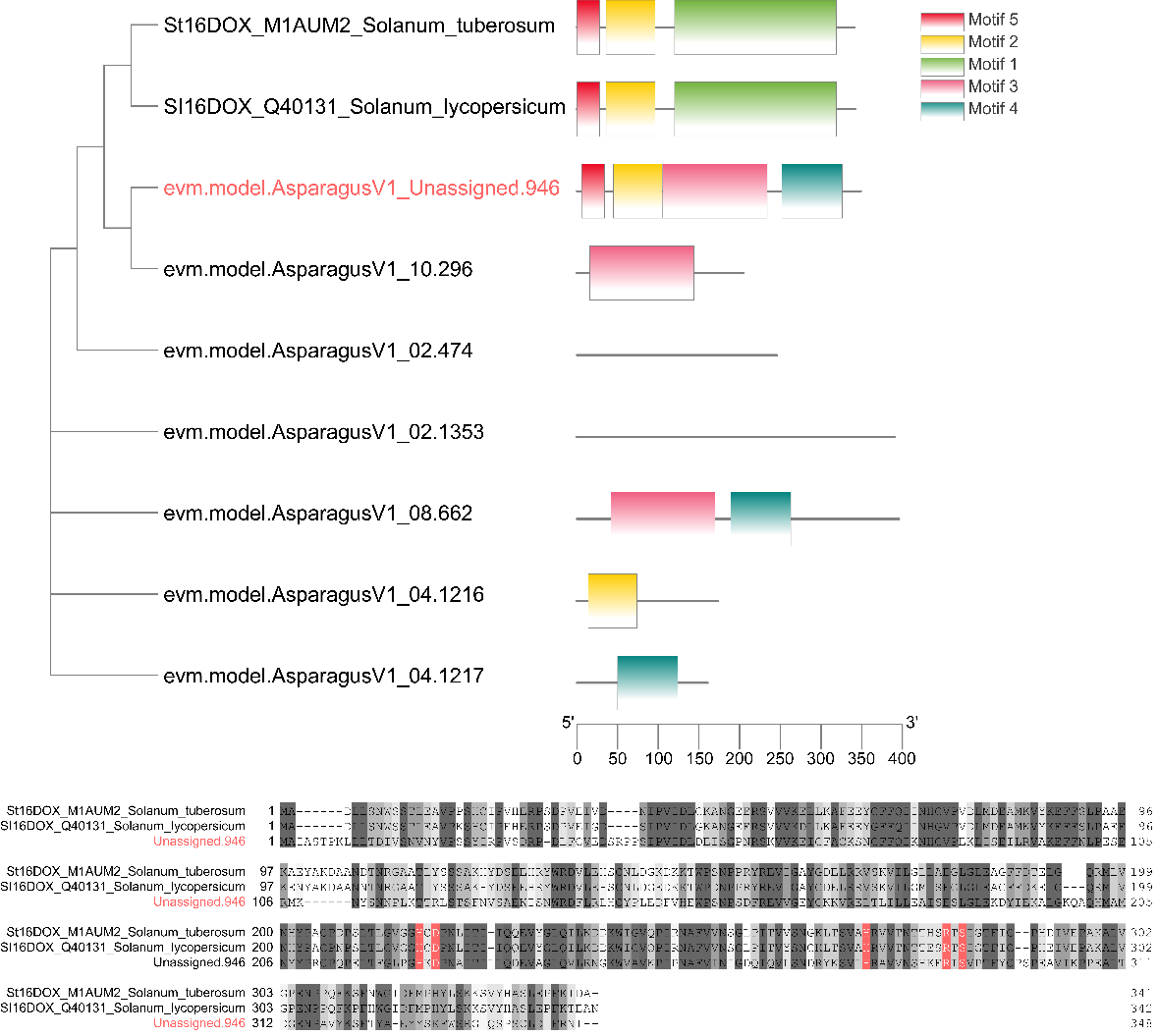


Fig S8. Identification of C16 hydroxylase in asparagus. The tree was constructed using TBtools with protein motifs predicted by MMEM, and the MSA was constructed with 16DOXs of potato and tomato using Jalviwe. The key domains were heightened in red.


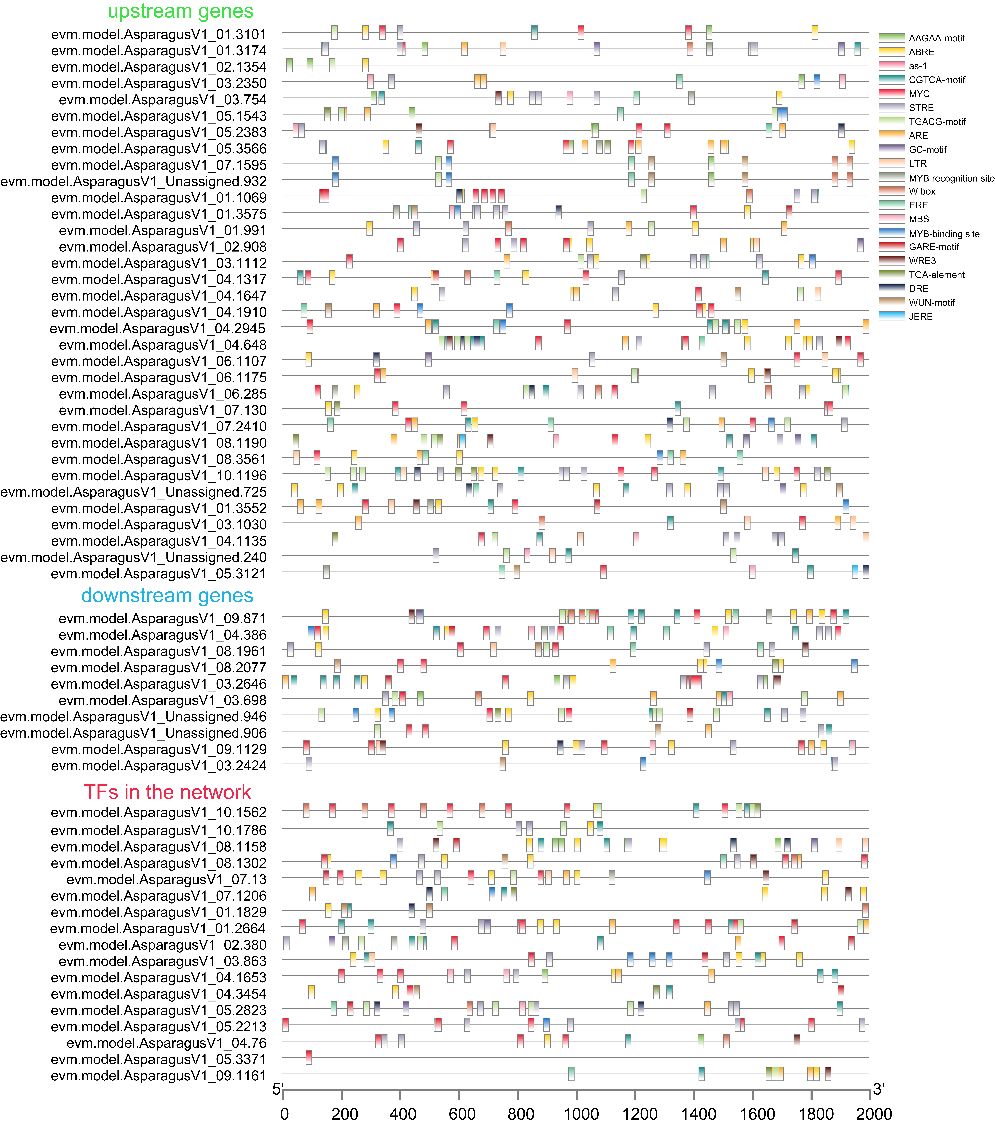


Fig S9. The cis-active elements prediction of all steroid-related genes’ and TFs’ promoters was performed using TBtools.


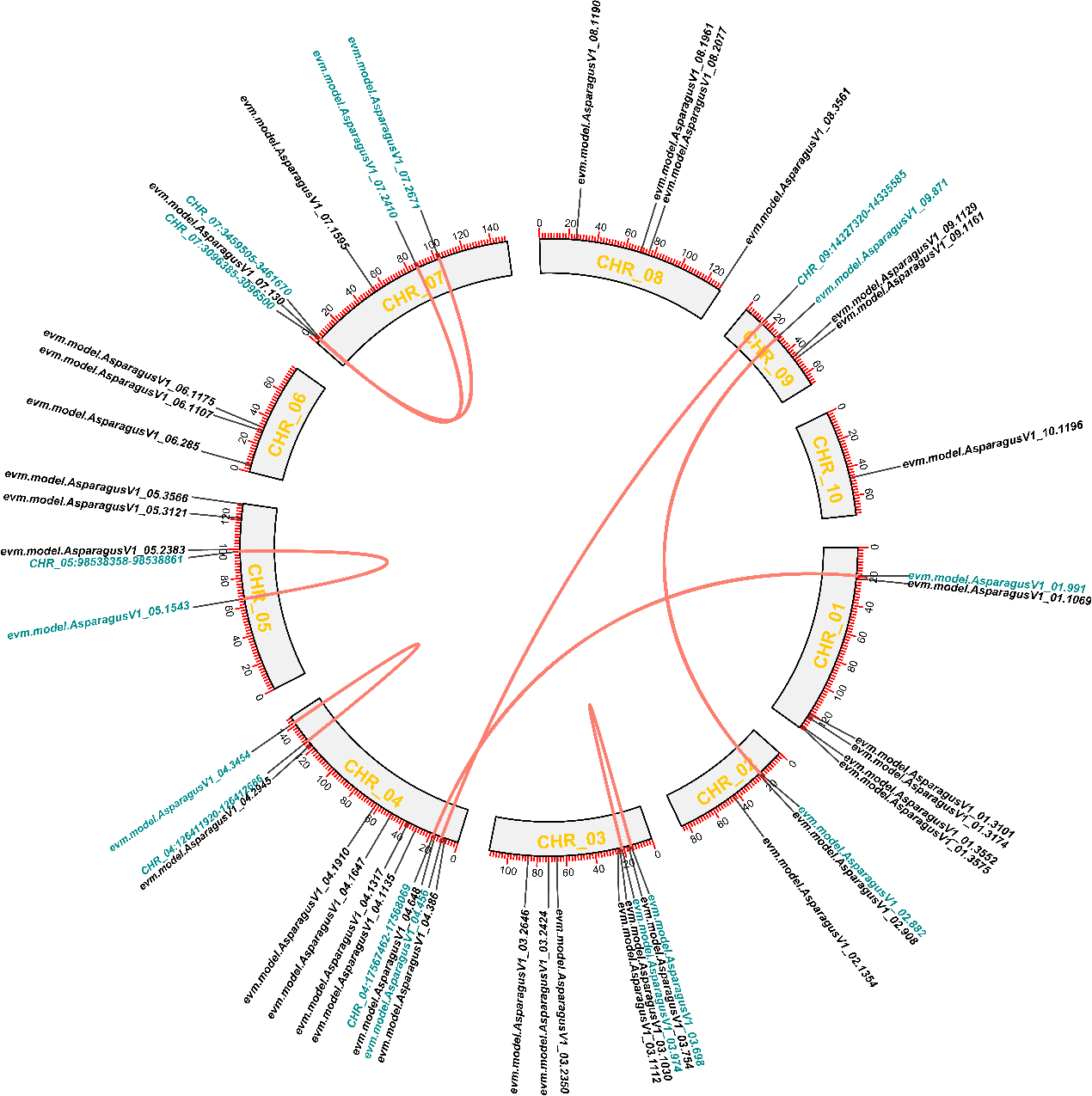


Fig S10 The chromosome location and fusions genes of SSP detected with Pacbio Isoseq data. The fused genes were highlighted in red linker with the blue ids.
