# Supplementary material for: Steroidal saponin profiles and their key genes for synthesis and regulation in Asparagus officinalis L. by joint analysis of metabolomics and transcriptomics
Source: BMC Plant Biol. 2023 Apr 20;23:207. doi: 10.1186/s12870-023-04222-x (PMC10116787; doi:10.1186/s12870-023-04222-x)
Supplement: Supplementary file 3 — Additional file 3. [file 12870_2023_4222_MOESM3_ESM.docx]

Secondary mass spectrum of 18 steroid and 2 terpenoid metabolites

C01 Trillin (Diosgenin-3-O-glucoside)


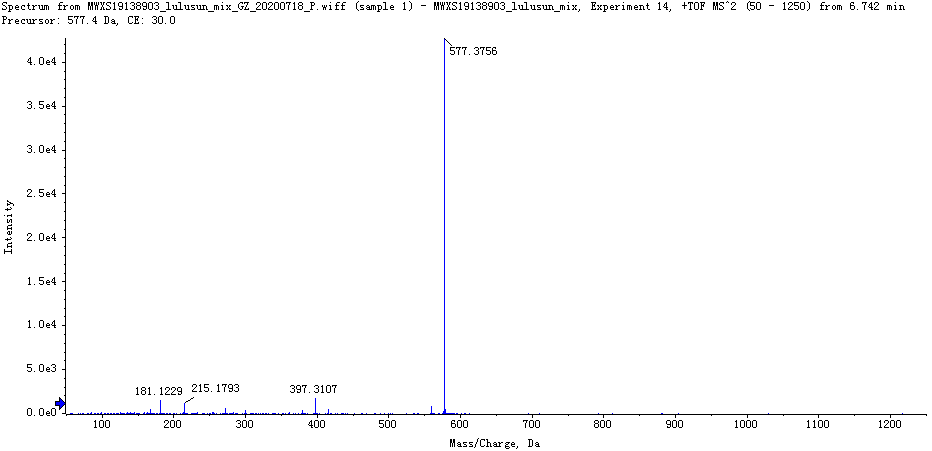


C02 Diosgenin-3-O-rhamnosyl(1,2)glcoside


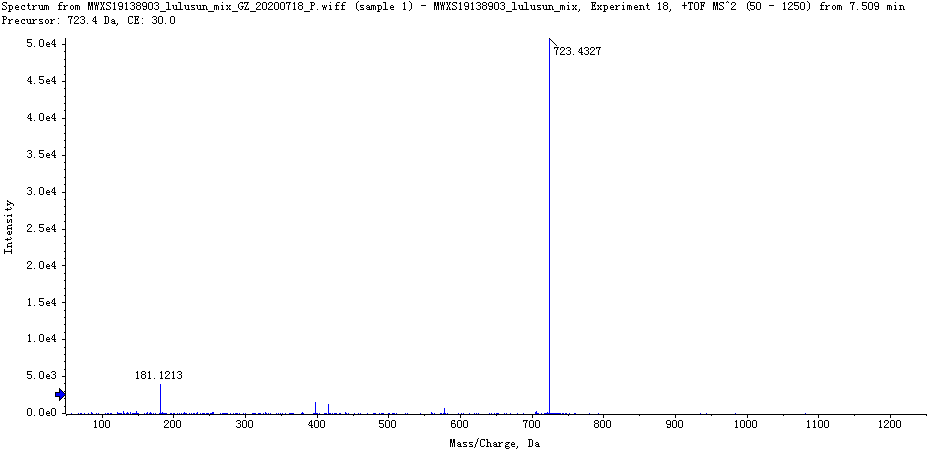


C03 HydroxyDiosgenin-rhamnosyl(1,2)glucoside


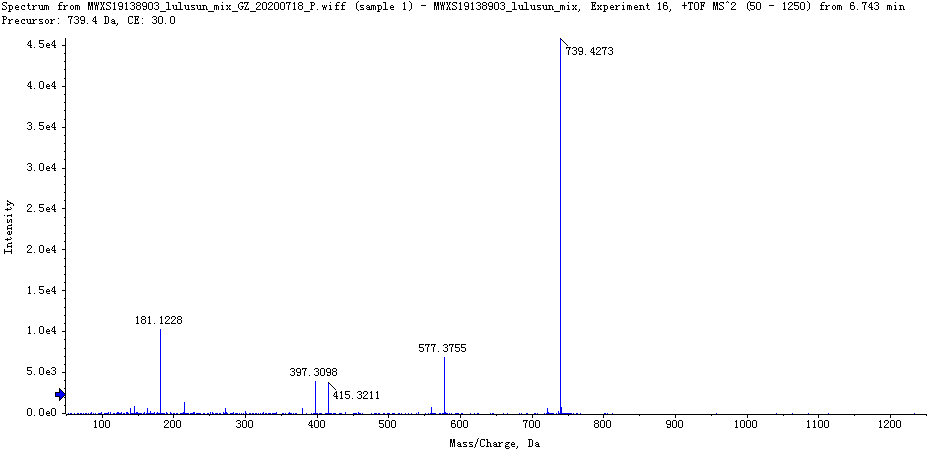


C04 Dioscin


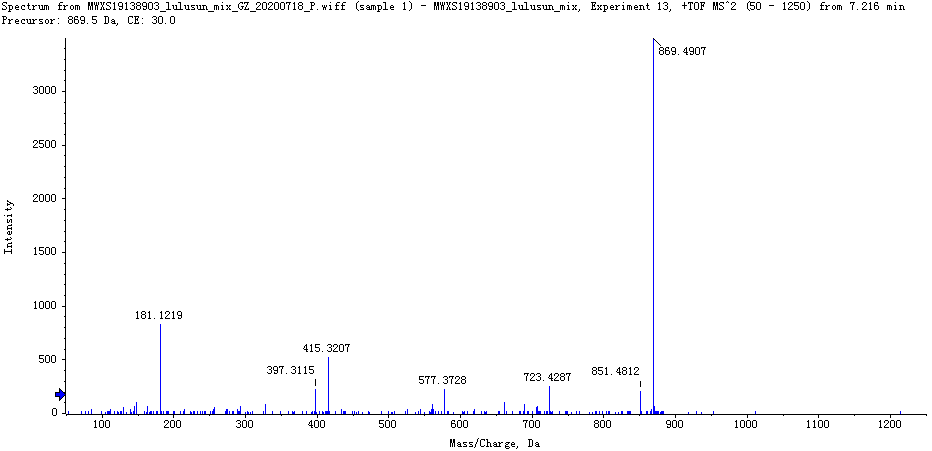


C05 Diosgenin rha-glc-glc


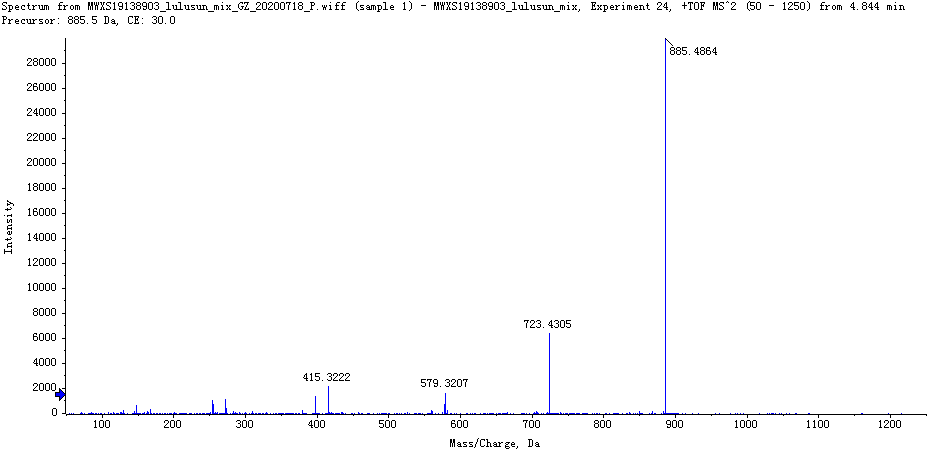


C06 Pseudoprotodioscin


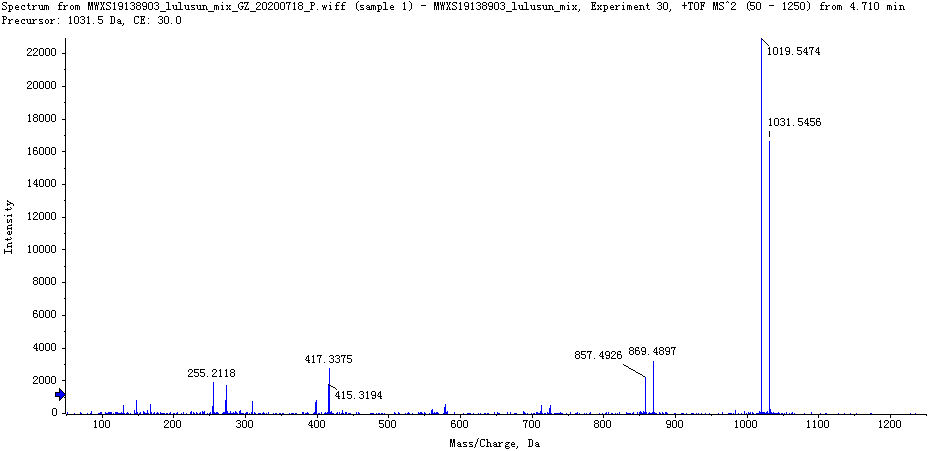


C07 Protodioscin


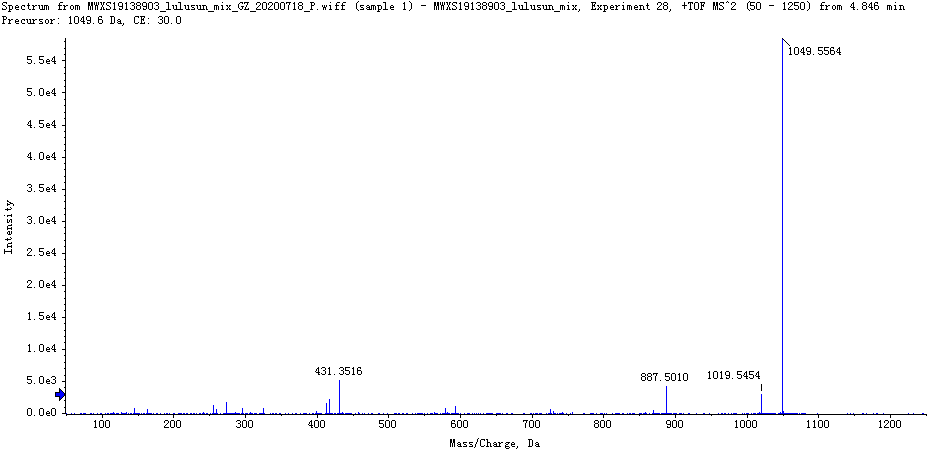


C08 Trillin-6'-O-sophorotrioside


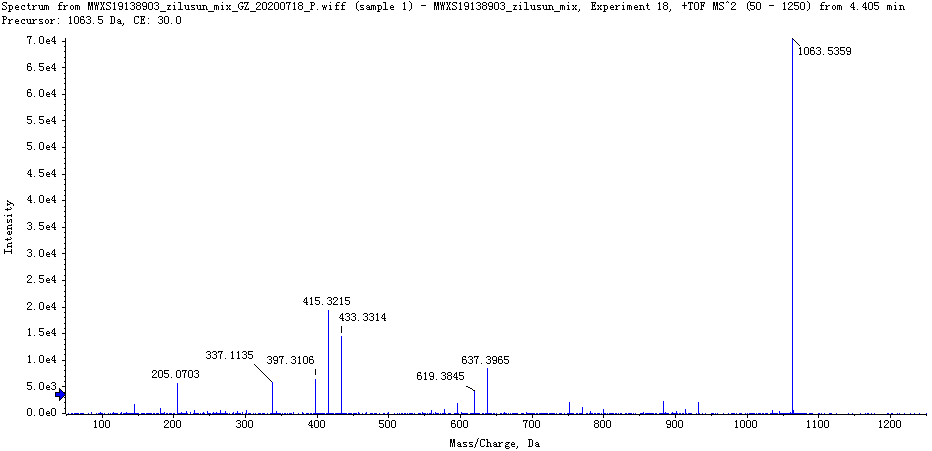


C09 Sweroside


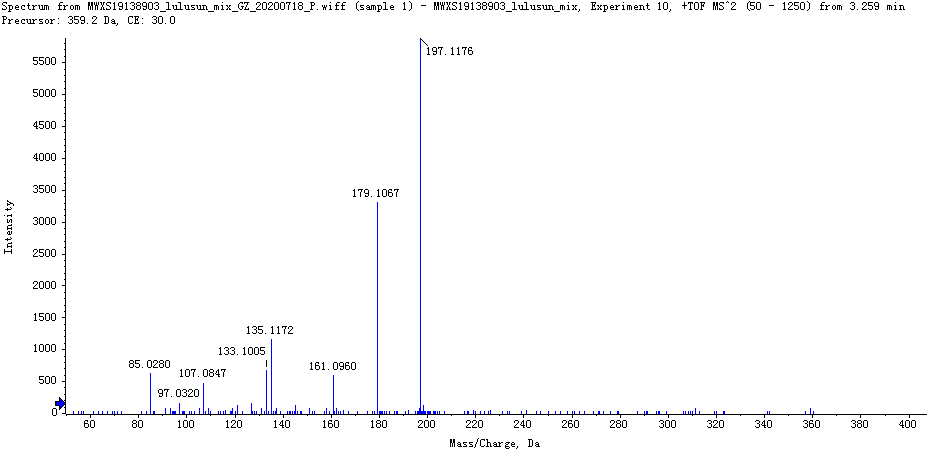


C10 Cholesterol


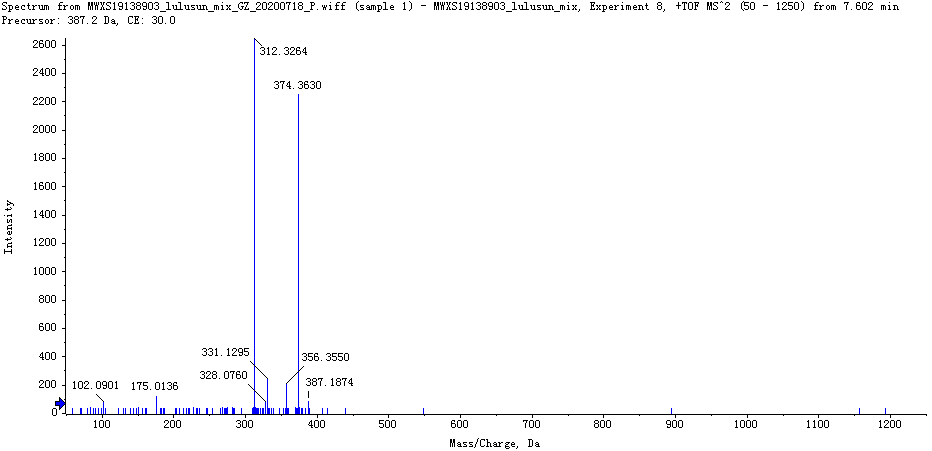


C11 Diosgenin


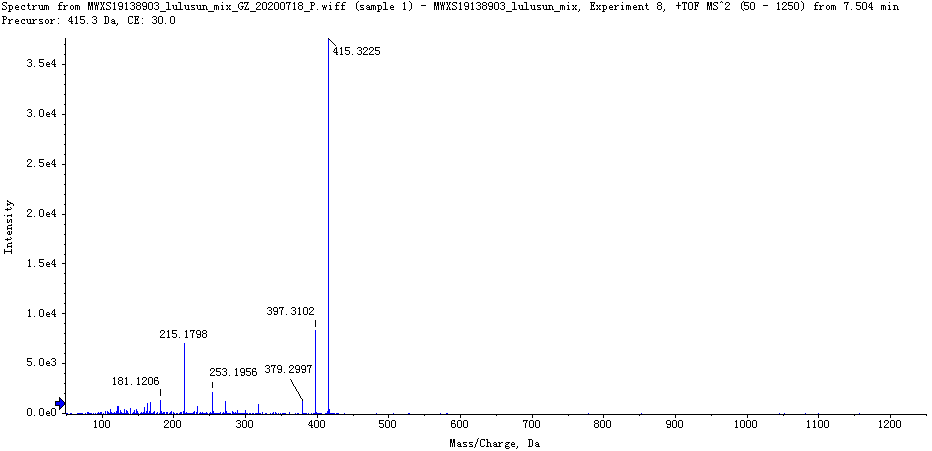


C12 Markogenin


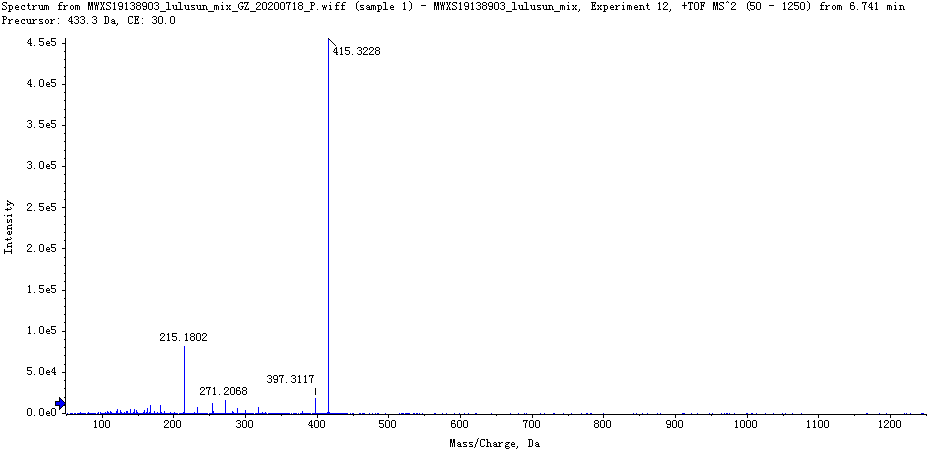


C13 Podecdysone C


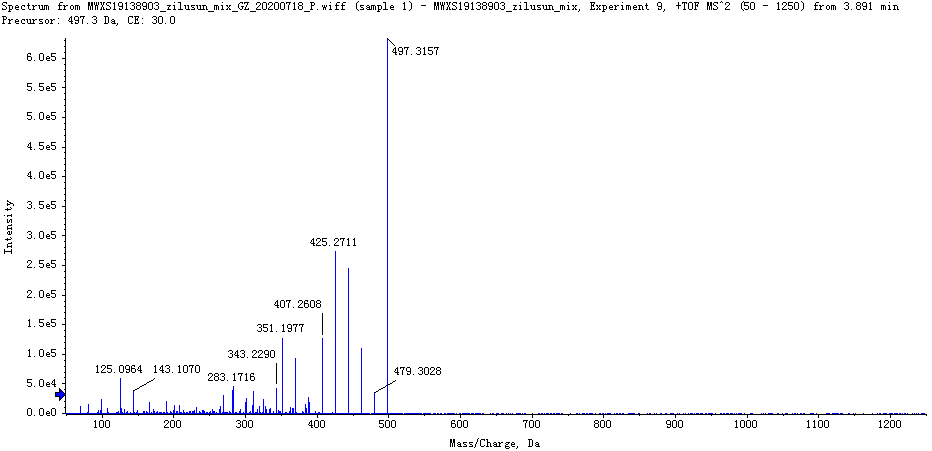


C14 3β-alcohol-5-β progesterone-16-ene-20-one-3-O-a-L-arabinopyranosyl


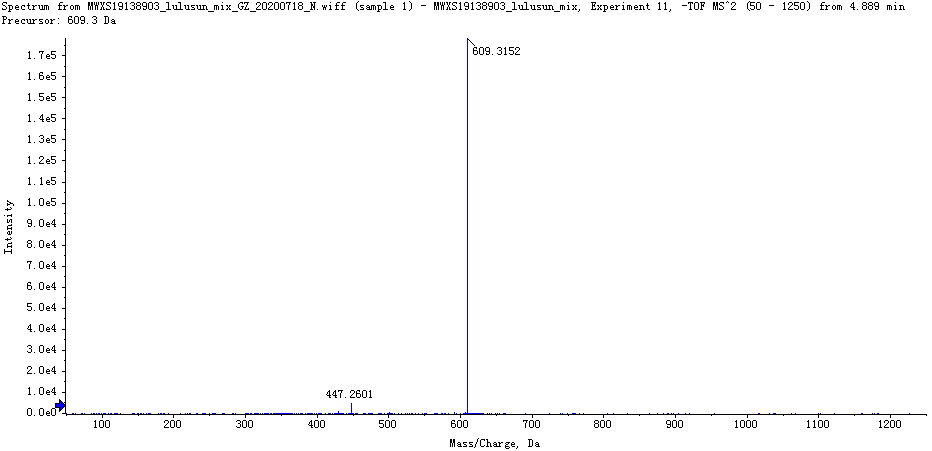


C15 (25S) -5-β-spirosteroid-3-β-ol-3-O-a-L-rhamnoside (1,4) -β-D-glucosyl


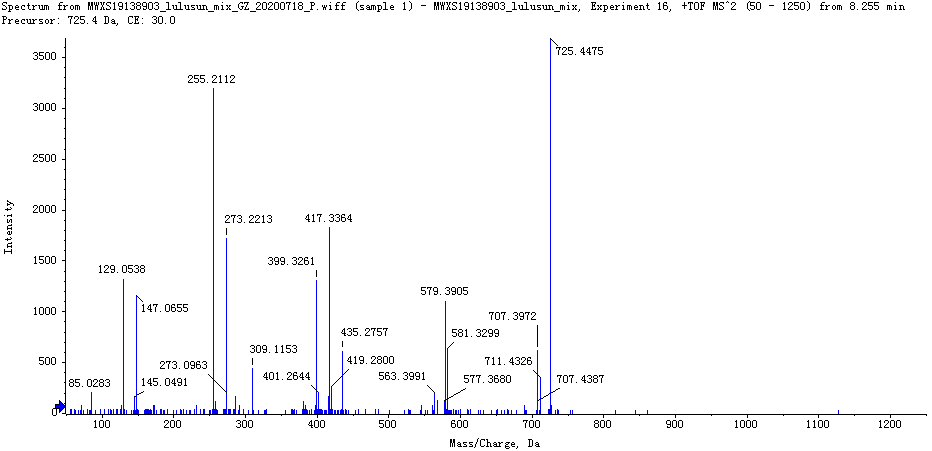


C16 Asparagoside C


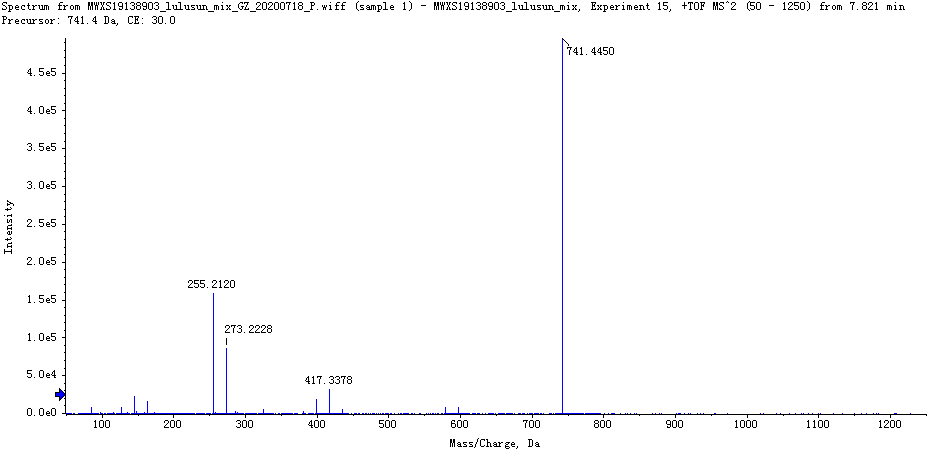


C17 asparanin B


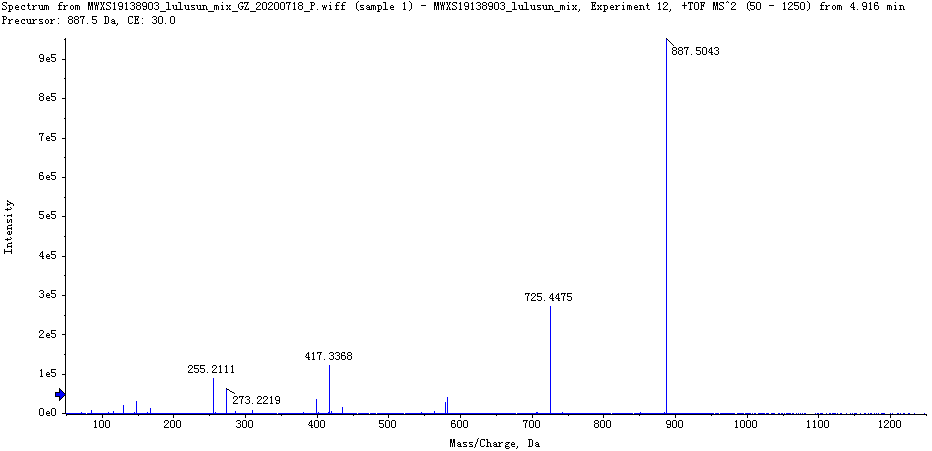


C18 Asparasaponin II


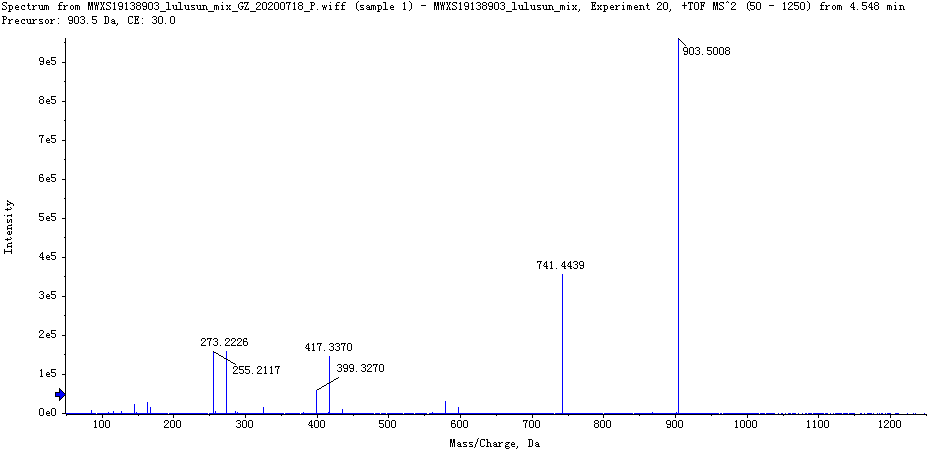


C19 (25S) -26-O-β-D-glucosyl-5β-furostone-3β, 22α, 26-triol-3-O-α-L-rhamnyl (1,4) β-D- Glucosyl


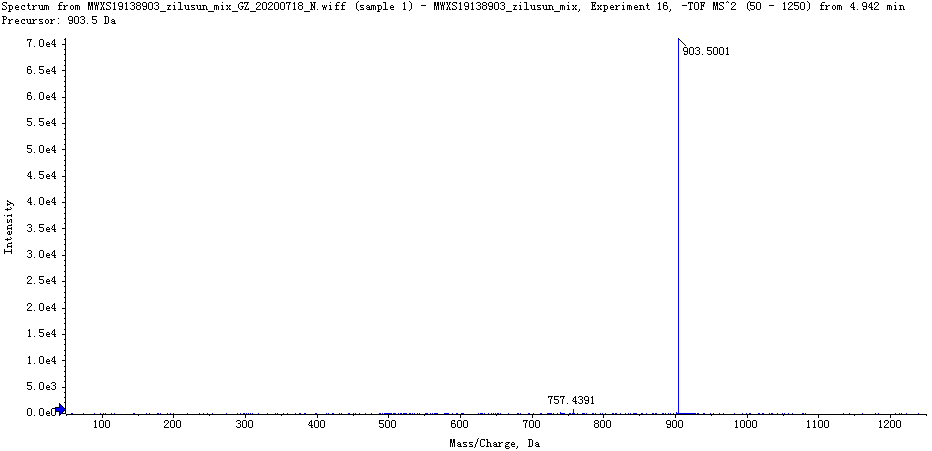


C20 Asparagoside F


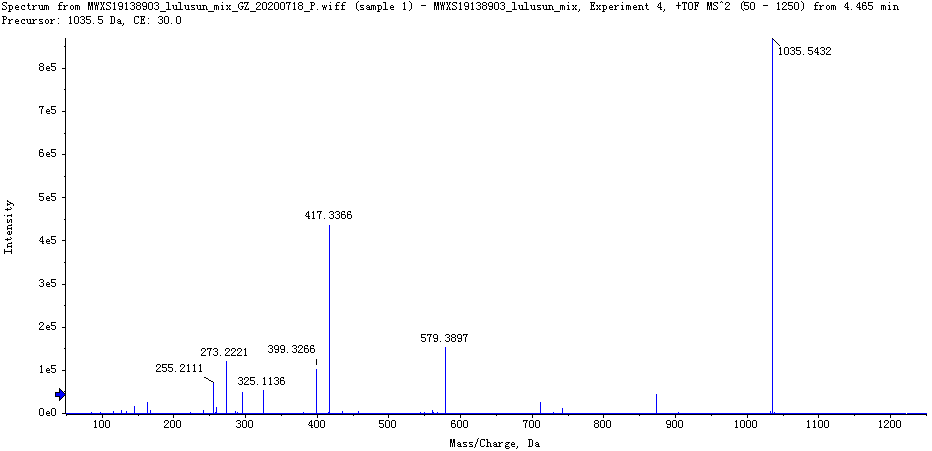


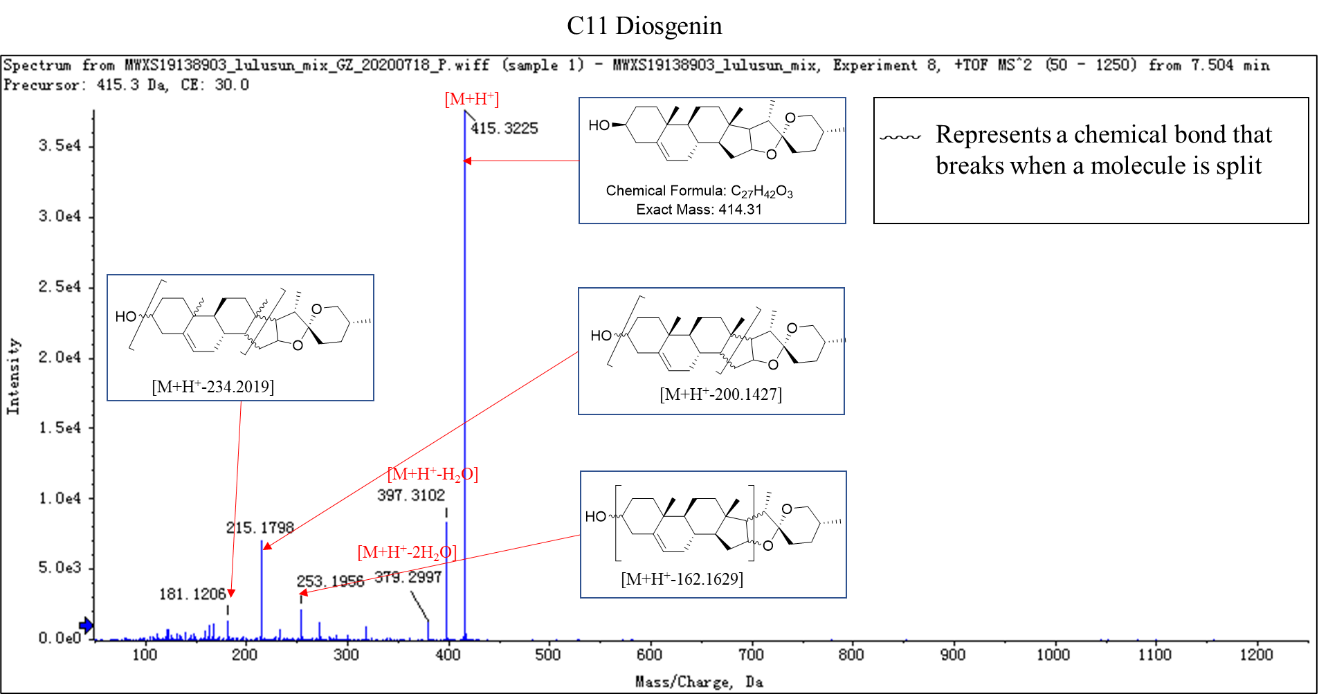


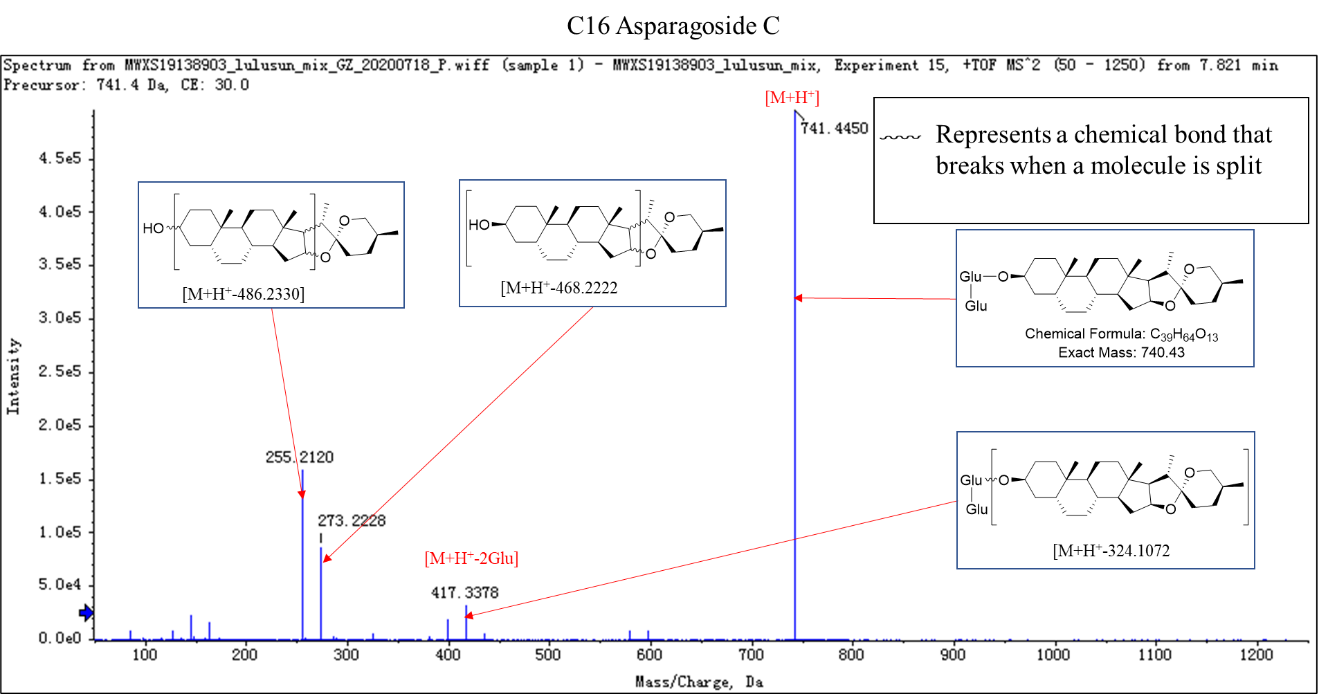


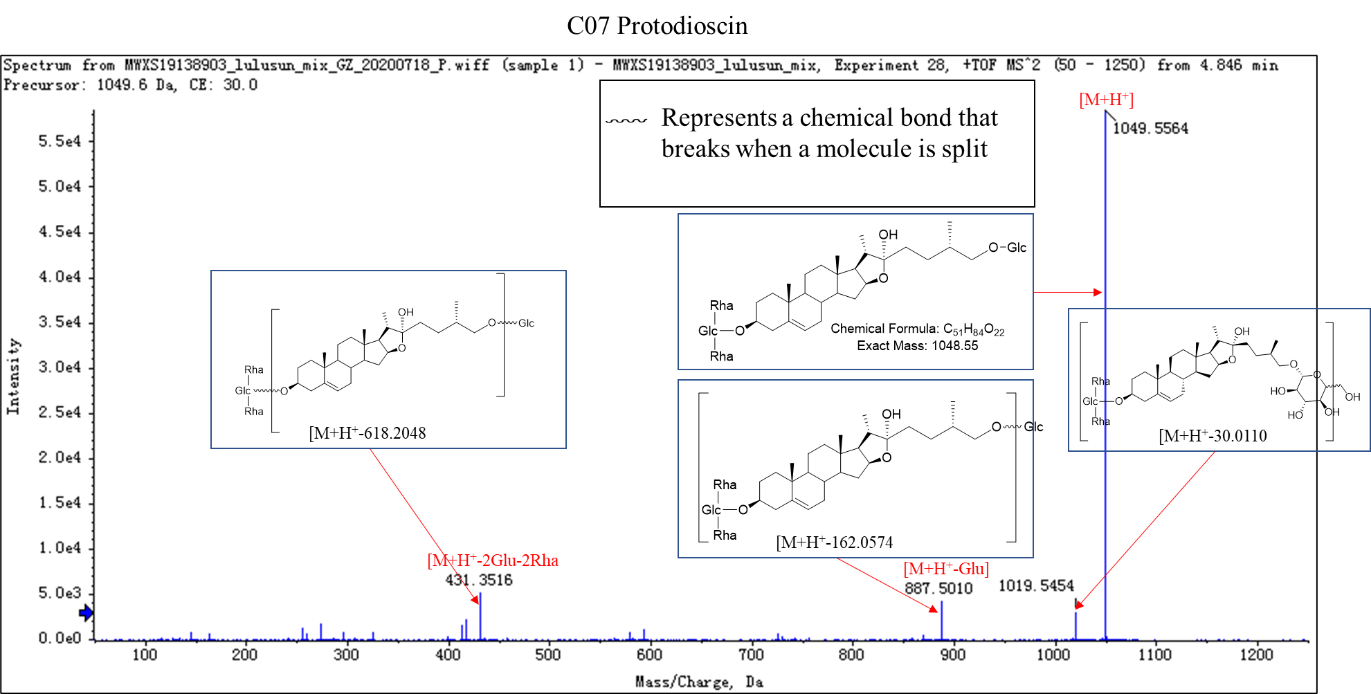


| **The qualitative basis of steroidal metabolites** | | | | | | | | | | | | | | | | | | | |
| --- | --- | --- | --- | --- | --- | --- | --- | --- | --- | --- | --- | --- | --- | --- | --- | --- | --- | --- | --- |
| **Compounds** | **No.** | **type** | **Q1 (Da)** | **Q3 (Da) (Characteristic fragment ion peak of secondary mass spectrometry)** | | | | | | | | | | | **Rt (min)** | **Molecular Weight (Da)** | **Formula** | **Ionization model** | **Level** |
| Trillin (Diosgenin-3-O-glucoside) | C01 | Isospirosteroid | 577.37 | 181.12 | 215.18 | - | - | 397.31 | - | - | - | - | - | - | 6.84 | 576.36 | C33H52O8 | [M+H]+ | A |
| Diosgenin-3-O-rhamnosyl(1,2)glcoside | C02 | Isospirosteroid | 723.43 | 181.12 | - | - | - | 397.30 | - | - | - | - | - | - | 7.62 | 722.42 | C39H62O12 | [M+H]+ | A |
| HydroxyDiosgenin-rhamnosyl(1,2)glucoside | C03 | Isospirosteroid | 739.42 | 181.12 | - | - | - | 397.31 | 415.32 | - | - | 577.38 | - | - | 6.85 | 738.42 | C39H62O13 | [M+H]+ | A |
| Dioscin | C04 | Isospirosteroid | 869.48 | ` | - | - | - | 397.31 | 415.32 | - | - | 577.37 | 723.43 | - | 7.31 | 868.48 | C45H72O16 | [M+H]+ | A |
| Diosgenin rha-glc-glc | C05 | Isospirosteroid | 885.47 | - | - | - | - | - | 415.32 | - | - | 579.32 | 723.43 | - | 4.87 | 884.48 | C45H72O17 | [M+H]+ | A |
| Pseudoprotodioscin | C06 | Furostanes | 1031.54 | - | - | 255.21 | - | - | 415.32 | 417.34 | - | - | - | 1019.55 | 4.74 | 1030.54 | C51H82O21 | [M+H]+ | A |
| Protodioscin | C07 | Furostanes | 1049.55 | - | - | - | - | - | - | - | 431.35 | - | - | 1019.55 | 4.89 | 1048.55 | C51H84O22 | [M+H]+ | A |
| Trillin-6'-O-sophorotrioside | C08 | Isospirosteroid | 1063.53 | - | - | - | - | 397.31 | 415.32 | - | 433.33 | - | - | - | 4.38 | 1062.52 | C51H82O23 | [M+H]+ | A |
| Sweroside | C09 | - | 359.13 |  | - | - | - | - | - | - | - | - | - | - | 3.33 | 358.13 | C16H22O9 | [M+H]+ | B |
| Cholesterol | C10 | - | 387.10 | - | - | - | - | 331.10 | - | - | - | - | - | - | 7.59 | 386.36 | C27H46O | [M+H]+ | B |
| Diosgenin | C11 | Isospirosteroid | 415.32 | 181.12 | 215.18 | 253.20 | 289.21 | 397.31 | - | - | - | - | - | - | 7.32 | 414.31 | C27H42O3 | [M+H]+ | A |
| Markogenin | C12 | Spirosteroid | 433.33 | - | 215.18 | - | 271.21 | 397.31 | 415.32 | - | - | - | - | - | 6.89 | 432.32 | C27H44O4 | [M+H]+ | B |
| Podecdysone C | C13 | - | 497.31 | - | - | - | - | 351.20 | - | - | - | - | - | - | 3.93 | 496.30 | C27H44O8 | [M+H]+ | A |
| 3β-alcohol-5-β progesterone-16-ene-20-one-3-O-a-L-arabinopyranosyl | C14 | - | 609.31 | - | - | - | - | 447.26 | - | - | - | - | - | - | 4.89 | 610.34 | C32H50O11 | [M-H]- | A |
| (25S) -5-β-spirosteroid-3-β-ol-3-O-a-L-rhamnoside (1,4) -β-D-glucosyl | C15 | Spirosteroid | 725.38 | - | - | 255.21 | 273.22 | 399.33 | - | 417.34 | 435.28 | 579.31 | - | - | 8.00 | 724.44 | C39H64O12 | [M+H]+ | A |
| Asparagoside C | C16 | Spirosteroid | 741.44 | - | - | 255.21 | 273.22 | - | - | 417.34 | - | - | - | - | 7.92 | 740.44 | C39H64O13 | [M+H]+ | A |
| asparanin B | C17 | Spirosteroid | 887.51 | - | - | 255.21 | 273.22 | - | - | 417.34 | - | - | 725.45 | - | 4.93 | 886.49 | C45H74O17 | [M+H]+ | A |
| Asparasaponin II | C18 | Furostanes | 903.49 | - | - | 255.21 | 273.22 | 399.33 | - | 417.34 | - | - | 741.44 | - | 4.56 | 902.49 | C45H74O18 | [M+H]+ | A |
| (25S) -26-O-β-D-glucosyl-5β-furostone-3β, 22α, 26-triol-3-O-α-L-rhamnyl (1,4) β-D- Glucosyl | C19 | Furostanes | 903.50 | - | - | - | - | - | - | - | - | - | 757.43 | - | 4.94 | 904.50 | C45H76O18 | [M-H]- | A |
| Asparagoside F | C20 | Spirosteroid | 1035.54 | - | - | 255.21 | 273.22 | 399.33 | - | 417.34 | - | 579.37 | - | - | 4.47 | 1034.53 | C50H82O22 | [M+H]+ | A |
